# Activity-dependent post-translational regulation of palmitoylating and depalmitoylating enzymes in the hippocampus

**DOI:** 10.1242/jcs.260629

**Published:** 2023-04-11

**Authors:** Danya Abazari, Angela R. Wild, Tian Qiu, Bryan C. Dickinson, Shernaz X. Bamji

**Affiliations:** ^1^ Department of Cellular and Physiological Sciences, Life Sciences Institute and Djavad Mowafaghian Centre for Brain Health, University of British Columbia, Vancouver, BC, V6T 1Z3, Canada; ^2^ Department of Chemistry, University of Chicago, Chicago, IL 60637, USA

**Keywords:** Palmitoylation, ZDHHC enzymes, Chemical long-term potentiation, Synapse activity, Post-translational modification

## Abstract

Activity-induced changes in protein palmitoylation can regulate the plasticity of synaptic connections, critically impacting learning and memory. Palmitoylation is a reversible post-translational modification regulated by both palmitoyl-acyl transferases that mediate palmitoylation and palmitoyl thioesterases that depalmitoylate proteins. However, it is not clear how fluctuations in synaptic activity can mediate the dynamic palmitoylation of neuronal proteins. Using primary hippocampal cultures, we demonstrate that synaptic activity does not impact the transcription of palmitoylating and depalmitoylating enzymes, changes in thioesterase activity, or post-translational modification of the depalmitoylating enzymes of the ABHD17 family and APT2 (also known as LYPLA2). In contrast, synaptic activity does mediate post-translational modification of the palmitoylating enzymes ZDHHC2, ZDHHC5 and ZDHHC9 (but not ZDHHC8) to influence protein–protein interactions, enzyme stability and enzyme function. Post-translational modifications of the ZDHHC enzymes were also observed in the hippocampus following fear conditioning. Taken together, our findings demonstrate that signaling events activated by synaptic activity largely impact activity of the ZDHHC family of palmitoyl-acyl transferases with less influence on the activity of palmitoyl thioesterases.

## Teaser

**Highlighted Article:** Activity-induced palmitoylation of synaptic proteins is important for synapse plasticity and is mediated through post-translational modification of the ZDHHC palmitoylating enzymes.

## INTRODUCTION

The formation and remodeling of synaptic contacts requires the precise distribution and trafficking of proteins to specialized compartments. Although post-translational phosphorylation of synaptic proteins has been well studied and shown to play a key role in regulating synaptic plasticity (Anggono and Huganir, 2012; Esteban et al., 2003; Hayashi, 2021; Lee, 2006; Lua et al., 2010; Man et al., 2003; Woolfrey and Dell'Acqua, 2015), more recent studies have demonstrated that other post-translational modifications, including protein *S*-acylation, can be equally important for the strengthening and weakening of synaptic connections (Matt et al., 2019; Ji and Skup, 2021; Fukata and Fukata, 2010).

The most prominent form of *S*-acylation, and the most common post-translational lipid modification in the brain, is *S*-palmitoylation (hereafter called palmitoylation). Palmitoylation involves the reversible addition of palmitoyl moieties to selected cysteine residues via thioester bonds, increasing protein hydrophobicity and the affinity for plasma membranes. This reaction is catalyzed by a family of 23 palmitoylating zinc-finger DHHC-domain-containing (ZDHHC) enzymes and reversed by a subset of depalmitoylating enzymes in the serine hydrolase superfamily (Main and Fuller, 2022). Approximately 41% of all known synaptic proteins can be palmitoylated (Sanders et al., 2015), including ion channels (Pei et al., 2018), SNARE proteins (Greaves et al., 2010; He and Linder, 2009), scaffold proteins (El-Husseini et al., 2000; Purkey et al., 2018; Topinka and Bredt, 1998; Vallejo et al., 2017), signaling molecules (Rocks et al., 2005) and neurotransmitter receptors, including AMPA, NMDA and GABA receptor subunits (Hayashi et al., 2009; 2005; Lin et al., 2009; Resh, 2006; Thomas and Huganir, 2013).

Notably, several studies have shown that synaptic proteins can be differentially palmitoylated in response to synaptic activity and that the dynamic palmitoylation of synaptic proteins is essential for synapse plasticity. Using an unbiased proteomic approach, our laboratory has identified 121 proteins (56 synaptic proteins) that are differentially palmitoylated in response to fear conditioning, and that a subset of these proteins is also differentially palmitoylated in response to increased synaptic activity in primary hippocampal cultures (Nasseri et al., 2022). Moreover, work from our laboratory and others have shown that increased synaptic activity can increase the palmitoylation of PSD-95 (or DLG4) (Noritake et al., 2009), AKAP79/150 (AKAP5) (Keith et al., 2012; Woolfrey et al., 2015), δ-catenin (CTNND1) (Brigidi et al., 2015; 2014) and plasticity-related gene 1 (PRG-1; also known as lipid phosphate phosphatase-related protein type 4 or LPRR4) (Nasseri et al., 2022), and that the palmitoylation of these proteins is essential for the recruitment and retention of AMPA receptors (AMPARs) to the synaptic membrane and the strengthening of synaptic connections. Although these studies demonstrate that dynamic protein palmitoylation is important for synaptic plasticity, it is unclear how changes in synaptic activity can alter protein palmitoylation.

According to BrainPalmSeq, an RNA-sequencing database tool developed in our laboratory, the majority of ZDHHC enzymes are expressed in hippocampal excitatory neurons (Wild et al., 2022). Although many ZDHHCs reside within the somatic Golgi apparatus where they constitutively palmitoylate proteins, several are localized to dendrites where they can locally and dynamically palmitoylate synaptic proteins. These include ZDHHC2 (Fukata et al., 2013), ZDHHC5 (Brigidi et al., 2015; Thomas et al., 2012), ZDHHC8 (Thomas et al., 2012), ZDHHC9 (Shimell et al., 2019), ZDHHC14 (Sanders et al., 2020) and ZDHHC15 (Shah et al., 2019). Although identification of the full family of depalmitoylating enzymes is still underway, a subset is known to be expressed in neurons (Wild et al., 2022) and localized to neuronal processes. These include APT1 (or LYPLA1) and APT2 (LYPLA2) (Milde and Coleman, 2014; Shen et al., 2022), PPT1, which is targeted to axons (Ahtiainen et al., 2003; Kim et al., 2008), and the more recently discovered α/β-hydrolase domain-containing protein 17 members (ABHD17A, ABHD17B and ABHD17C; collectively referred to as ABHD17), which have numerous post-synaptic substrates, including PSD-95, BK channels and N-RAS (Lin and Conibear, 2015; McClafferty et al., 2020; Yokoi et al., 2016). These palmitoylating and depalmitoylating enzymes are, therefore, well positioned to mediate dynamic substrate palmitoylation that occurs following changes in synaptic activity.

Although increasing evidence suggests that synaptic activity leads to the differential palmitoylation of neuronal proteins and that palmitoylation is important for the strengthening and weakening of synaptic connections, the mechanisms by which this occurs are largely unknown. In this study, we demonstrate that activity-induced changes in protein palmitoylation are largely driven by the dynamic regulation of palmitoylating enzymes as opposed to depalmitoylating enzymes. Increasing synaptic activity in primary hippocampal cultures mediates the post-translational modification of the palmitoylating enzymes ZDHHC2, ZDHHC5 and ZDHHC9, but not of ZDHHC8 or the depalmitoylating enzymes APT2 and ABHD17. We further demonstrate that these post-translational modifications are essential for ZDHHC enzyme stability, protein interactions as well as enzymatic activity. Notably, similar changes in the post-translational modifications of these ZDHHC enzymes occurred 1 h after fear conditioning, highlighting the importance of ZDHHC modifications in regulating synapse function *in vivo*. Taken together, these data suggest that the differential palmitoylation of synaptic proteins upon synaptic stimulation is mediated by post-translational modifications of ZDHHC enzymes, which in turn regulate enzyme stability and function.

## RESULTS

### ZDHHC enzyme transcription is largely unchanged after increased synaptic activity

**Fig. 1.**
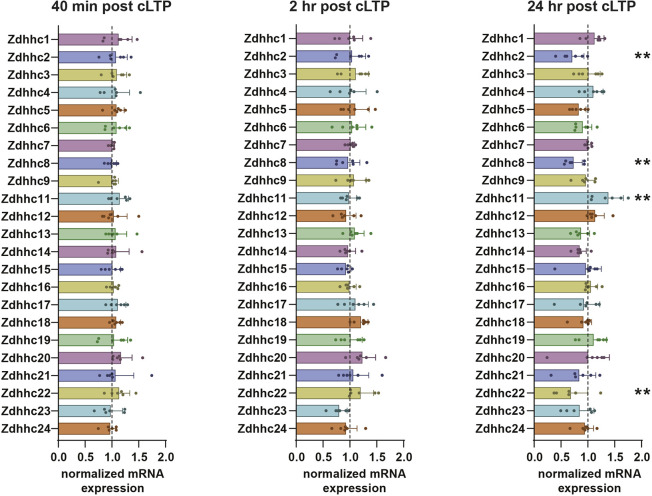
**ZDHHC mRNA levels are not changed 40** **min and 2** **h post cLTP.** qRT-PCR of the 23 ZDHHC enzymes from primary hippocampal cultures 40 min, 2 h or 24 h following cLTP. Values are normalized to mock-cLTP treated control. **P<0.01 (unpaired two-tailed Student's *t*-test versus mock-cLTP control). Results are mean±s.e.m. with individual data points shown. *n=*6 independent hippocampal cultures per condition.

Strong evidence from multiple studies has shown that changes in synaptic activity can dramatically alter the transcriptional profiles of neuronal proteins (Flavell and Greenberg, 2008; West et al., 2002). Notably, protocols that induce long-term potentiation (LTP) can also alter transcription of numerous neuronal genes (Bliim et al., 2019; Tyssowski et al., 2018). To determine whether synaptic activity alters the transcriptional profile of ZDHHC enzymes as a means to regulate dynamic substrate palmitoylation, we increased synaptic activity in cultured hippocampal neurons using a well-established chemical LTP (cLTP) protocol involving a brief 3 min incubation with 200 μM glycine in the absence of Mg^2+^ (Lu et al., 2001), and quantified mRNA transcripts using quantitative real-time PCR (qRT-PCR) for the 23 ZDHHC enzymes (UniProt reviewed) 40 min, 2 h and 24h later. Although cLTP did not significantly alter transcription of the majority of the ZDHHCs at each time point (Fig. 1), expression of *Zdhhc2*, *Zdhhc8* and *Zdhhc22* was significantly reduced, and expression of *Zdhhc11* increased 24 h following cLTP induction. As activity-induced changes in protein palmitoylation have been shown to occur at much earlier time points (Brigidi et al., 2015; Nasseri et al., 2022; Noritake et al., 2009), it is unlikely that changes in the transcription of these few ZDHHC genes are grossly responsible for alterations in protein palmitoylation. We therefore next investigated alternative regulatory mechanisms that might alter ZDHHC function minutes to hours after synaptic stimulation, when activity-induced changes in substrate palmitoylation are known to occur (Nasseri et al., 2022).

### ZDHHC antibody validation for biochemical study of endogenous ZDHHCs

To assay activity-induced changes in endogenous ZDHHCs, we first needed to identify commercially available antibodies that detected ZDHHCs with high specificity. Upon testing available antibodies for all 23 ZDHHC enzymes, good antibodies were identified for ZDHHC2, ZDHHC5, ZDHHC6, ZDHHC8 and ZDHHC9 (Fig. S1). We chose to further study ZDHHC2 and ZDHHC5 as they have been shown to regulate activity-induced palmitoylation of synaptic proteins (Brigidi et al., 2015, 2014; Fukata et al., 2013; Noritake et al., 2009), as well as ZDHHC8 and ZDHHC9 as their function is disrupted in a subset of patients with schizophrenia (Mukai et al., 2004) and X-linked intellectual disability (Baker et al., 2015; Raymond et al., 2007), respectively. Notably, these four enzymes are highly expressed in hippocampal neurons (Wild et al., 2022), have well-defined roles in regulating synaptic function, and localize to neuronal dendrites and spines where they are appropriately positioned to mediate rapid, dynamic changes in the palmitoylation of synaptic proteins in response to changes in synaptic activity (Brigidi et al., 2015; Fukata et al., 2013; Shimell et al., 2021; 2019; Thomas et al., 2013; 2012; Woolfrey and Dell'Acqua, 2015).

### Activity-dependent ZDHHC5 degradation is regulated by phosphorylation of a polo-box motif

**Fig. 2.**
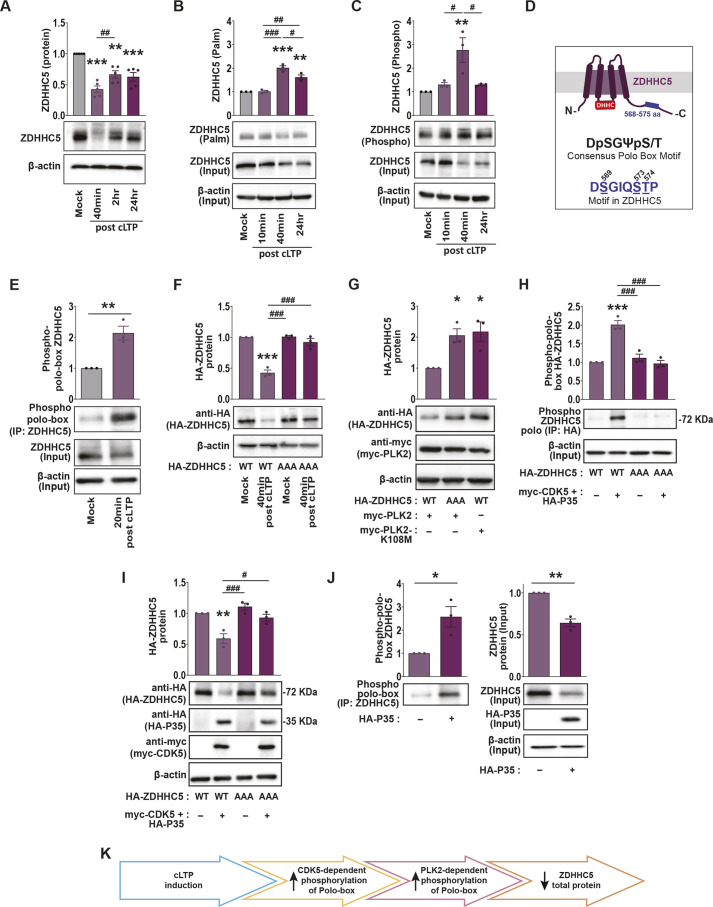
**cLTP-induced phosphorylation of ZDHHC5 polo-box domain impacts ZDHHC5 stability.** (A) Western blot analysis of ZDHHC5 protein levels in primary hippocampal neuron cultures 40 min, 2 h and 24 h following cLTP. *n=*5 independent hippocampal cultures per condition. (B) Acyl-Rac assay showing palmitoylated (‘palm’) ZDHHC5 and overall ZDHHC5 protein (input) levels following cLTP. Palmitoylated ZDHHC5 values in the graph were derived from ZDHHC5 ‘palm’ normalized to ZDHHC5 ‘input’ and the β-actin loading control. *n=*3 independent hippocampal cultures per condition. (C) Lysates were run through the phospho-protein purification assay and western blots probed with the anti-ZDHHC5 antibody showing phosphorylated ZDHHC5 and overall ZDHHC5 protein (input) levels following cLTP. Phosphorylated ZDHHC5 values in the graph were derived from ZDHHC5 ‘phospho’ normalized to ZDHHC5 ‘input’ and the β-actin loading control. *n=*3 independent hippocampal cultures per condition. (D) Schematic of the sequence and position of the polo-box motif within the C-terminal tail of ZDHHC5. (E) Hippocampal culture lysates were immunoprecipitated (IP) with an anti-ZDHHC5 antibody and western blots probed with a phospho-polo-box-specific antibody. *n=*3 independent hippocampal cultures per condition. (F) Cells were nucleofected with ZDHHC5 shRNA to knock down ZDHHC5, and with HA–ZDHHC5-WT or the HA–ZDHHC5-AAA mutant at the time of plating. At 13–15 DIV, the cultures were stimulated using cLTP treatment and HA–ZDHHC5 protein levels determined using western blot analysis. *n=*3 independent hippocampal cultures per condition. (G) Cells were nucleofected with myc–PLK2 -WT or kinase-dead PLK2 (myc–PLK2-K108M) together with HA–ZDHHC5-WT or HA–ZDHHC5-AAA. ZDHHC5 or PLK2 protein levels were determined at 14 DIV by western blotting. *n=*3 independent hippocampal cultures per condition. (H) Cells were nucleofected with HA–ZDHHC5-WT or HA–ZDHHC5-AAA together with myc–CDK5 and HA–P35. At 13–15 DIV, HA-tagged proteins were immunoprecipitated (IP) with an anti-HA antibody and western blots probed with a phospho-polo-box-specific antibody to determine ZDHHC5 polo-box phosphorylation. *n=*3 independent hippocampal cultures per condition. (I) Cells were nucleofected with HA–ZDHHC5-WT or HA–ZDHHC5-AAA together with myc–CDK5 and HA–P35. At 14 DIV, western blotting was performed to assay the protein levels of all transfected constructs. *n=*3 independent hippocampal cultures per condition. (J) Cells were nucleofected with HA–P35. At 14 DIV, endogenous ZDHHC5 was immunoprecipitated (IP) with an anti-ZDHHC5 antibody and western blots were probed with a phospho-polo-box antibody to assay ZDHHC5 polo-box phosphorylation (left). The input from this assay was run on a western blot and probed for endogenous ZDHHC5 and HA–P35 (right). *n=*3 independent hippocampal cultures per condition. For all graphs, results are mean±s.e.m. with individual data points shown. *P<0.05; **P<0.01; ***P<0.001; determined versus the first condition in the bar chart (unpaired two-tailed Student's *t*-test). ^#^*P*<0.05; ^##^*P*<0.01; ^###^*P*<0.001; pairwise comparison as indicated (one-way ANOVA with Tukey's post hoc test). (K) Schematic of activity-dependent changes in ZDHHC5 C-terminal polo-box regulation of ZDHHC5 total protein.

Our laboratory has previously shown that synaptic activity increases palmitoylation of the cadherin-binding protein, δ-catenin, and that this is mediated by the dephosphorylation of ZDHHC5 on tyrosine residue 533 and the subsequent internalization of ZDHHC5 from the plasma membrane (Brigidi et al., 2015). To get a more fulsome understanding of how synaptic activity can impact ZDHHC5 function, we monitored cLTP-induced changes in protein turnover and post-translational modifications. We focused on the post-translational modifications phosphorylation and palmitoylation, which are both highly dynamic and have considerable influence over synaptic protein function and localization (Ji and Skup, 2021; Lee, 2006). Furthermore, kinases and phosphatases that mediate phosphorylation are highly responsive to synaptic activity (Woolfrey and Dell'Acqua, 2015), whereas certain ZDHHCs are known to engage in palmitoylation cascades wherein ZDHHC enzymes are themselves palmitoylation substrates for other ZDHHC enzymes that control their function (Abrami et al., 2017; Plain et al., 2020). We observed a substantial (>50%) reduction in the total protein levels of ZDHHC5 40 mins post-cLTP, which recovered slightly at 2 h but did not return to baseline levels after 24 h (Fig. 2A). We also observed an activity-dependent increase in the palmitoylation of ZDHHC5 using an acyl resin-assisted capture (acyl-Rac) assay (Badrilla, UK) at 40 mins and 24 h post cLTP, after the palmitoylated fraction was normalized to total ZDHHC5 levels (Fig. 2B). Additionally, when using a phospho-protein affinity enrichment assay (PhosphoProtein Purification Kit; QIAGEN) to assess the overall change in the amount of phosphorylated ZDHHC5, we observed a significant overall increase in the phosphorylated fraction of ZDHHC5 (when normalized to total protein input; Fig. 2C). As this increase in ZDHHC5 phosphorylation initially appeared to be counter to our previous observations of decreased tyrosine phosphorylation following cLTP (Brigidi et al., 2015), we further investigated which phospho-residues might be responsible for the overall net increase in ZDHHC5 phosphorylation and how this might be related to the substantial decrease in total ZDHHC5 protein.

Using bioinformatics analysis of the C-terminal region of ZDHHC5, we identified a sequence (DSGIQSTP) very similar to the consensus polo-box motif DpSGΨXpS/T (Ψ represents a hydrophobic residue, X is any residue, pS or pS/T indicates phosphorylated serine or threonine), so named due to the sequence being recognized by the polo domain present in polo-like kinases (Nakojima et al., 2003; Fig. 2D). When dually phosphorylated on serine/threonine residues, this motif targets proteins for rapid ubiquitination and degradation (Ang et al., 2008; Arai et al., 2008; Moshe et al., 2004; Pak and Sheng, 2003; Seeburg et al., 2008). We therefore investigated whether cLTP increases ZDHHC5 phosphorylation on these serine/threonine residues and whether this regulates ZDHHC5 stability. Hippocampal culture lysates were immunoprecipitated with ZDHHC5 and blots probed with an antibody that specifically recognizes the phosphorylated polo-box motif (Baehr et al., 2016; Wang et al., 2018) 20 mins following cLTP treatment. We observed a dramatic 2-fold increase in the phosphorylation of serine/threonine residues in this motif, despite a significant decrease in ZDHHC5 protein levels (input) in neurons treated with cLTP (Fig. 2E). To further ascertain whether the phosphorylation of this motif is required for activity-induced degradation of ZDHHC5, hippocampal neurons were transfected with ZDHHC5 shRNA to knockdown endogenous ZDHHC5, along with HA-tagged wild-type (WT) ZDHHC5 (HA–ZDHHC5-WT) or phospho-dead ZDHHC5 (HA–ZDHHC5-AAA), in which Ser569, Ser573 and Thr574 in the polo-box motif were changed to alanines. Although cLTP significantly decreased the expression of HA–ZDHHC5-WT, phospho-dead HA–ZDHHC5-AAA levels were unchanged (Fig. 2F), demonstrating that phosphorylation of this motif is required for degradation of ZDHHC5 following cLTP.

We next set out to determine how synaptic activity can regulate ZDHHC5 phosphorylation and subsequently the destabilization of ZDHHC5. Previous studies have shown that polo-like kinase 2 (PLK2) can phosphorylate residues within polo-box motifs (Ang et al., 2008; Lee et al., 2011). We therefore determined whether PLK2 is involved in phosphorylation-dependent degradation of ZDHHC5. Although overexpression of myc-tagged WT PLK2 (myc–PLK2-WT) resulted in a decrease in HA–ZDHHC5-WT protein levels, it had no effect on HA–ZDHHC5-AAA total protein levels (Fig. 2G). Moreover, the PLK2 kinase-dead mutant, in which Lys108 is mutated to a methionine (myc–PLK2-K108M), did not impact HA–ZDHHC5-WT protein levels (Fig. 2G), demonstrating that PLK2 mediates ZDHHC5 degradation through phosphorylation of the polo-box motif.

Prior to phosphorylation by PLK2, DpSGΨXpS/T-containing peptides have shown to be phosphorylated by proline-directed kinases such as cyclin dependent kinases (CDKs) (Hamanaka et al., 1995; Martin and Strebhardt, 2006; Seeburg et al., 2008; Thomas et al., 2016). Indeed, it is thought that CDK-mediated phosphorylation can prime proteins to be phosphorylated by PLK2 (Elia et al., 2003). Previous work has identified CDK5 as the priming kinase that phosphorylates serine–threonine–proline (STP) motifs in the substrate protein SPAR (Seeburg et al., 2008). To see whether CDK5 is involved in polo-box phosphorylation and the destabilization of ZDHHC5, hippocampal neurons were transfected with HA–ZDHHC5-WT or HA–ZDHHC5-AAA together with CDK5 and its neuronal-specific activator P35 (or CDK5R1) (Chae et al., 1997). Overexpression of myc–CDK5 and HA–P35 increased the phosphorylation of the polo-box domain (Fig. 2H) and decreased overall levels of HA–ZDHHC5-WT but not of HA–ZDHHC5-AAA (Fig. 2I). Finally, overexpression of HA–P35 alone was sufficient to activate endogenous CDK5 and increase phosphorylation of the polo-box motif within immunoprecipitated endogenous ZDHHC5 (Fig. 2J). This was accompanied by a decrease in total endogenous ZDHHC5 protein in the input fraction (Fig. 2J, right). We have therefore identified a mechanism by which PLK2, CDK5 and P35 regulate the stability of ZDHHC5 in neurons following synaptic activity (Fig. 2K). In line with our previous work, these results reveal that ZDHHC5 is highly responsive to synaptic activity and, as such, well positioned to mediate dynamic changes in palmitoylation of synaptic proteins.

### ZDHHC8 phosphorylation, palmitoylation and protein turnover are not affected by cLTP

**Fig. 3.**
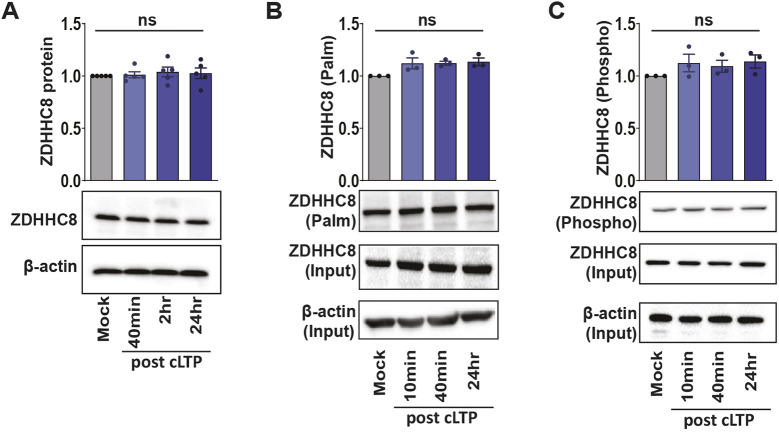
**ZDHHC8 protein levels, palmitoylation and phosphorylation are unchanged following cLTP.** (A) Western blot analysis of ZDHHC8 protein levels in primary hippocampal neuron cultures 40 min, 2 h and 24 h following cLTP. *n=*5 independent hippocampal cultures per condition. (B) Acyl-Rac assay showing palmitoylated ZDHHC8 and overall ZDHHC8 protein (input) levels following cLTP. Palmitoylated ZDHHC8 values in the graph were derived from ZDHHC8 ‘palm’ normalized to ZDHHC8 ‘input’ and the β-actin loading control. *n=*3 independent hippocampal cultures per condition. (C) Lysates were run through the phospho-protein purification assay and western blots probed with the anti-ZDHHC8 antibody showing phosphorylated ZDHHC8 and overall ZDHHC8 protein (input) levels following cLTP. Phosphorylated ZDHHC8 values in the graph were derived from ZDHHC8 ‘phospho’ normalized to ZDHHC8 ‘input’ and the β-actin loading control. ns, not significant (one-way ANOVA with Tukey's post hoc test). *n=*3 independent hippocampal cultures per condition. Results are mean±s.e.m. with individual data points shown.

We next investigated the effects of cLTP on the post-translational regulation of ZDHHC8, which has a role in synaptic development (Mukai et al., 2008) and was found to be disrupted in a subset of patients with schizophrenia (Mukai et al., 2004). ZDHHC8 localizes to dendritic projections and is the closest homologue of ZDHHC5, with which it shares 60% sequence similarity and 50% identity. Furthermore, ZDHHC8 contains many signaling motifs in common with ZDHHC5, including a C-terminal PDZ-binding domain (consisting of the residues EISV) (Thomas et al., 2012), tyrosine endocytic motif (YDNL) (Brigidi et al., 2015), along with a polo-box-like domain (DSGVYDT). We therefore determined whether ZDHHC8 post-translational modifications and protein turnover were altered by synaptic stimulation with cLTP. Surprisingly, no changes were observed in endogenous ZDHHC8 total protein levels 40 mins, 2 h and 24 h after cLTP (Fig. 3A), indicating that the reduction in ZDHHC8 mRNA we observed 24 h post cLTP (Fig. 1) did not significantly alter total protein turnover. We next investigated whether ZDHHC8 palmitoylation is modified by cLTP, as cysteine residues 236 and 245 of the murine ZDHHC8 C-terminal region have been shown to be palmitoylated (Collins et al., 2017). Accordingly, we detected palmitoylated ZDHHC8 in the palmitoylated fraction using the acyl-Rac assay, but this was not altered following cLTP (Fig. 3B). Finally, we did not observe changes in ZDHHC8 phosphorylation following cLTP treatment (Fig. 3C). Taken together, these results indicate that unlike ZDHHC5, ZDHHC8 might be less responsive to increased synaptic activity stimulated by cLTP, despite its localization to neuronal dendrites and synapses (Thomas et al., 2012).

### ZDHHC9 palmitoylation and substrate palmitoylation are decreased by cLTP

**Fig. 4.**
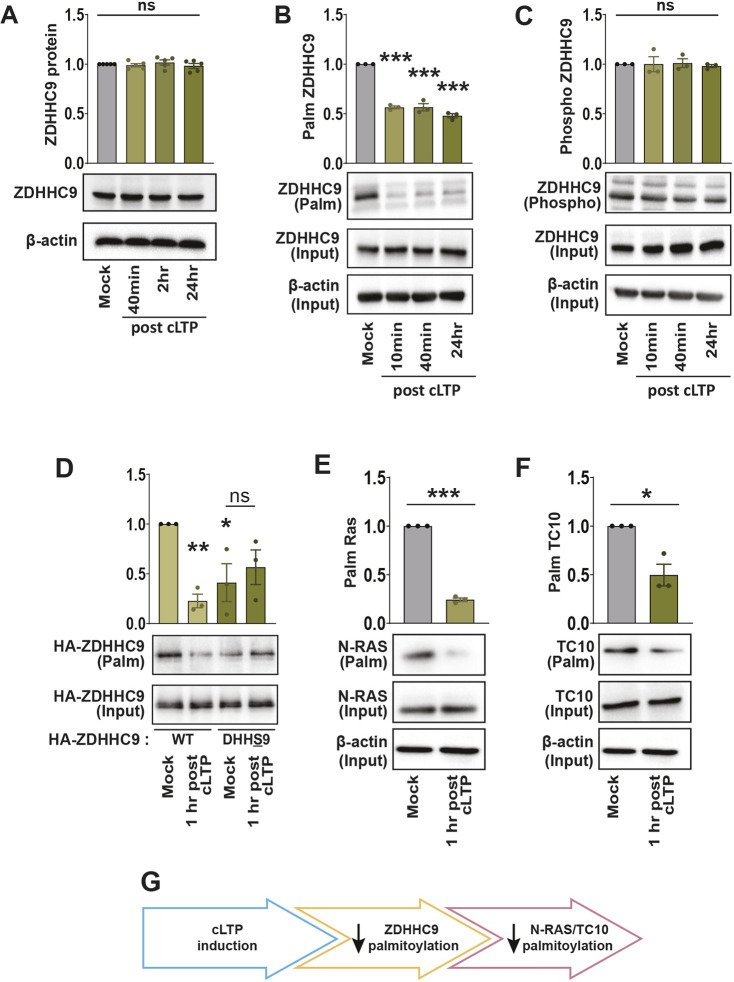
**The palmitoylation of ZDHHC9 and its substrates are reduced following cLTP.** (A) Western blot analysis of ZDHHC9 protein levels in primary hippocampal neuron cultures 40 min, 2 h and 24 h following cLTP. *n=*5 independent hippocampal cultures per condition. (B) Acyl-Rac assay showing palmitoylated ZDHHC9 and overall ZDHHC9 protein (input) levels following cLTP. Palmitoylated ZDHHC9 values in the graph were derived from ZDHHC9 ‘palm’ normalized to ZDHHC9 ‘input’ and the β-actin loading control. *n=*3 independent hippocampal cultures per condition. (C) Lysates were run through the phospho-protein purification assay and western blots probed with the anti-ZDHHC9 antibody showing phosphorylated ZDHHC9 and overall ZDHHC9 protein (input) levels following cLTP. Phosphorylated ZDHHC9 values in the graph were derived from ZDHHC9 ‘phospho’ normalized to ZDHHC9 ‘input’ and the β-actin loading control. *n=*3 independent hippocampal cultures per condition. (D) Cells were nucleofected at the time of plating with ZDHHC9 shRNA to knock down ZDHHC9, and with HA–ZDHHC9-WT or HA–ZDHHC9-DHHS9. At 13–15 DIV, the cultures were stimulated using cLTP treatment and HA–ZDHHC9 palmitoylation was determined using the Acyl-Rac assay. *n=*3 independent hippocampal cultures per condition. (E,F) At 14 DIV, the cultures were stimulated using cLTP treatment and N-RAS (E) or TC10 (F) palmitoylation was determined using the Acyl-Rac assay. *n=*3 independent hippocampal cultures per condition. For all graphs, results are mean±s.e.m. with individual data points shown. ns, not significant; *P<0.05; **P<0.01; ***P<0.001. For A–D, *P*-values were determined versus the first condition in the bar chart (one-way ANOVA with Tukey's post hoc test). For E,F, *P*-values were determined using unpaired two-tailed Student's *t*-test. (G) Schematic showing activity-induced decrease in ZDHHC9 and substrate palmitoylation.

We recently demonstrated that disrupting ZDHHC9 function *in vitro* decreases both dendritic outgrowth and the formation of inhibitory synapses (Shimell et al., 2019). As above, we assayed the effects of cLTP on ZDHHC9 palmitoylation, phosphorylation and turnover. We found that although ZDHHC9 protein levels (Fig. 4A) and phosphorylation (Fig. 4C) were unchanged, palmitoylation of ZDHHC9 was significantly decreased to 50% of the baseline levels 10 min after cLTP and was maintained up to 24 h after cLTP treatment (Fig. 4B). Previous work has shown that ZDHHC enzymes are first palmitoylated on the cysteine residue in the DHHC domain before transferring palmitate (palmitic acid) to its substrate (Stix et al., 2020). To determine whether cLTP specifically decreases ZDHHC9 palmitoylation at this site, cells were transfected with either WT or a DHHC motif mutant (Cys169 mutated to serine; referred to as DHHS9). As expected, there was a decrease in the palmitoylation of WT ZDHHC9 1 h after cLTP (Fig. 4D). Basal palmitoylation of ZDHHC9-DHHS9 was significantly reduced compared to that of ZDHHC9-WT, demonstrating that palmitoylation of Cys169 does indeed contribute to the overall palmitoylation of ZDHHC9. Notably, ZDHHC9-DHHS9 palmitoylation did not decrease further following cLTP treatment, indicating that this residue is subject to activity-dependent depalmitoylation (Fig. 4D).

ZDHHC9 has several neuronal substrates including N-RAS and TC10 (or RHOQ) (Shimell et al., 2019). To determine whether activity-induced depalmitoylation of ZDHHC9 impacts the palmitoylation of N-RAS and TC10, we assayed their palmitoylation 1 h after cLTP induction. There was a substantial decrease in the palmitoylation of these two proteins (Fig. 4E,F), suggesting that synaptic activity can decrease ZDHHC9 enzymatic activity by reducing its palmitoylation within the catalytic domain (Fig. 4G).

### ZDHHC2 phosphorylation and decrease of its interaction with PSD-95 after cLTP

**Fig. 5.**
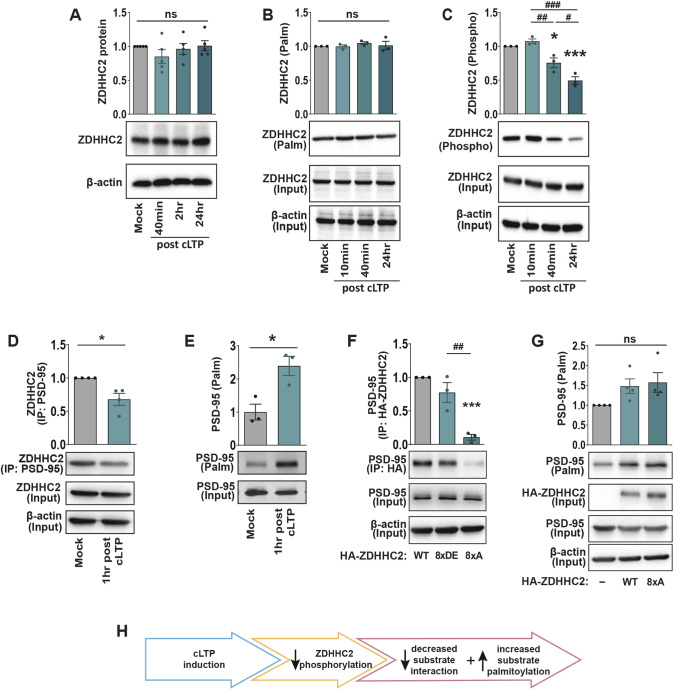
**Decreased phosphorylation of ZDHHC2 following cLTP.** (A) Western blot analysis of ZDHHC2 protein levels in primary hippocampal neuron cultures 40 min, 2 h and 24 h following cLTP. *n=*5 independent hippocampal cultures per condition. (B) Acyl-Rac assay showing palmitoylated ZDHHC2 and overall ZDHHC2 protein (input) levels following cLTP. Palmitoylated ZDHHC2 values in the graph were derived from ZDHHC2 ‘palm’ normalized to ZDHHC2 ‘input’ and the β-actin loading control. *n=*3 independent hippocampal cultures per condition. (C) Lysates were run through the phospho-protein purification assay and western blots probed with the anti-ZDHHC2 antibody showing phosphorylated ZDHHC2 and overall ZDHHC2 protein (input) levels following cLTP. Phosphorylated ZDHHC2 values in the graph were derived from ZDHHC2 ‘phospho’ normalized to ZDHHC2 ‘input’ and the β-actin loading control. *n=*3 independent hippocampal cultures per condition. *n=*3 independent hippocampal cultures per condition. (D) Hippocampal lysates were immunoprecipitated using an anti-PSD-95 antibody and western blots probed with an anti-ZDHHC2 antibody. The ZDHHC2 input was first normalized to β-actin as a loading control. The amount of the immunoprecipitated ZDHHC2 protein was then normalized to the β-actin-normalized input. *n=*4 independent hippocampal cultures per condition. (E) At 14 DIV, the cultures were stimulated using cLTP treatment and PSD-95 palmitoylation was determined using the Acyl-Rac assay. *n=*3 independent hippocampal cultures per condition. (F) HA–ZDHHC2-8×A phospho-dead mutation reduced co-immunoprecipitation of PSD-95. The PSD-95 input was first normalized to β-actin as a loading control. The amount of immunoprecipitated PSD-95 protein was then normalized to the β-actin normalized input. *n=*3 independent hippocampal cultures per condition. (G) Hippocampal cells were either left untransfected or transfected with HA–ZDHHC2-WT or HA–ZDHHC2-8×A at the time of plating. At 14 DIV, endogenous PSD-95 palmitoylation was determined using the Acyl-Rac assay. *n=*4 independent hippocampal cultures per condition. For all graphs, results are mean±s.e.m. with individual data points shown. *P<0.05; ***P<0.001; determined versus the first condition in the bar chart (unpaired two-tailed Student's *t*-test). ^#^*P*<0.05; ^##^*P*<0.01; ^###^*P*<0.001; pairwise comparison as indicated (one-way ANOVA with Tukey's post hoc test). (H) Schematic of activity-dependent changes in ZDHHC2 C-terminal phospho-regulation of PSD-95 substrate interactions.

ZDHHC2 localizes to hippocampal dendrites, where it cycles between recycling endosomes and the plasma membrane (Fukata et al., 2013). ZDHHC2 regulates the palmitoylation of the synaptic scaffold protein, PSD-95 (Fukata et al., 2013; Noritake et al., 2009), and AKAP79/150 (A-kinase anchoring protein; Woolfrey et al., 2015), thereby regulating the synaptic localization of AMPAR subunits and synapse strength. Similar to the other ZDHHCs as shown above, we interrogated activity-induced changes in ZDHHC2 protein turnover and post-translational modifications following cLTP treatment. Although total ZDHHC2 protein levels (Fig. 5A) and ZDHHC2 palmitoylation (Fig. 5B) were unchanged, we observed a significant reduction in the phosphorylation of ZDHHC2 40 min and 24 h post cLTP (Fig. 5C).

Synaptic activity can alter ZDHHC2-mediated palmitoylation of its downstream substrate, PSD-95 (Fukata et al., 2013). We observed a decrease in the association between ZDHHC2 and PSD-95 1 h after cLTP (Fig. 5D), which coincided with an increase in the palmitoylation of PSD-95 (Fig. 5E). Although these results initially appear to be contradictory, this could reflect changes in the kinetics of ZDHHC2 or changes in the binding domain of ZDHHC2 and/or PSD-95 after activity-induced ZDHHC2 dephosphorylation. To determine whether activity-induced changes in ZDHHC2/PSD-95 interactions and PSD-95 palmitoylation were directly due to changes in ZDHHC2 phosphorylation, we transfected cells with either ZDHHC2 phospho-mimetic [eight serine/threonine residues within the 330–366 amino acid (aa) region changed to aspartate/glutamate; HA–ZDHHC2-8×DE] or phospho-dead (eight serine/threonine residues within the 330–366 aa region changed to alanines; HA–ZDHHC2-8×A) (Salaun et al., 2017) constructs. These C-terminal serine and threonine residues were specifically targeted as phosphorylation of these sites has been shown to be important for membrane localization (Salaun et al., 2017) and as synaptic activity can alter the membrane localization of ZDHHC2 (Fukata et al., 2013). In line with our observed decrease in ZDHHC2 phosphorylation and ZDHHC2/PSD-95 interaction following cLTP, there was a robust decrease in the association of PSD-95 with the HA–ZDHHC2-8×A phospho-dead mutant compared to its association with HA–ZDHHC2-WT (Fig. 5F). The phospho-mimetic HA–ZDHHC2-8×DE mutant did not show higher association with PSD-95 compared to HA–ZDHHC2-WT, suggesting high basal phosphorylation of these serine and threonine residues (Fig. 5F). To determine whether decreased ZDHHC2 phosphorylation drove increased PSD-95 palmitoylation, we overexpressed HA–ZDHHC2-WT or the phospho-dead HA–ZDHHC2-8×A mutant. Both constructs increased the amount of palmitoylated PSD-95 equally (Fig. 5G), possibly because overexpression of either construct was sufficient to achieve saturated PSD-95 palmitoylation.

Overall, these results indicate that cLTP decreases the phosphorylation of ZDHHC2 at its C-terminal tail, resulting in a decrease in the association between PSD-95 and ZDHHC2 and an increase in PSD-95 palmitoylation (Fig. 5H).

### *In vivo* regulation of ZDHHCs following fear conditioning

**Fig. 6.**
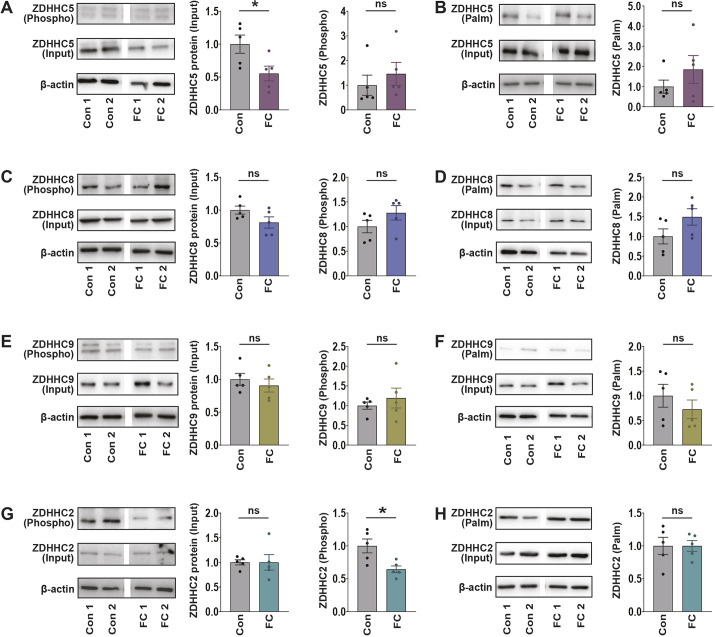
**ZDHHC activity-dependent post-translational changes *in vivo*.** Nine-week-old male mice were subjected to contextual fear conditioning (cFC) and hippocampal lysates from conditioned and unconditioned mice were collected 1 h later. (A,C,E,G) Lysates were run through the phospho-protein purification assay and western blots probed with antibodies for endogenous ZDHHC5, ZDHHC8, ZDHHC9 and ZDHHC2. Phosphorylated ZDHHC values in the graphs were derived from ZDHHC ‘phospho’ normalized to ZDHHC ‘input’ and the β-actin loading control. Input fractions were quantified to assess overall ZDHHC protein levels in control and cFC lysates. (B,D,F,H) Acyl-Rac assay showing palmitoylated ZDHHC5, ZDHHC8, ZDHHC9 and ZDHHC2 in control or cFC-treated mice. Palmitoylated ZDHHC values in the graphs were derived from ZDHHC ‘palm’ normalized to ZDHHC ‘input’ and the β-actin loading control. β-actin loading controls are duplicated in some panels as individual blots were cut in half and probed for two ZDHHCs per blot. Cut blots were probed for either ZDHHC2 (bottom half)/ZDHHC5 (top half) or ZDHHC9 (bottom half)/ZDHHC8 (top half). Con 1 and Con 2 represent two biological replicates from the control group. FC 1 and FC 2 represent two biological replicates from the cFC-treated group. ns, not significant; *P<0.05 (unpaired two-tailed Student's *t*-test). Results are mean±s.e.m. with individual data points shown. *n=*5 hippocampi per experiment.

Having identified a number of cLTP-induced post-translational modifications for ZDHHC enzymes *in vitro*, we next tested whether similar post-translational modifications occurred *in vivo* in response to a hippocampal-dependent learning event. Hippocampal lysates were collected 1 h after contextual fear conditioning (cFC) on adult male mice and changes in total protein, phosphorylation and palmitoylation were assayed (Fig. 6). In line with our *in vitro* results, we observed a significant decrease in ZDHHC5 protein levels after fear conditioning (input, Fig. 6A), in line with the activity-dependent decrease in ZDHHC5 protein stability observed after cLTP *in vitro* (Fig. 2A,B). However, this decrease was not accompanied by a relative increase in phosphorylation (Fig. 6A) or palmitoylation (Fig. 6B) as seen *in vitro*, perhaps due to differences in cell-type composition of the two samples or stimulation paradigms. No significant changes were observed for ZDHHC8 in any measurements following fear conditioning (Fig. 6C,D), in line with *in vitro* findings (Fig. 3). We did not observe changes in ZDHHC9 phosphorylation or palmitoylation following fear conditioning, indicating that the activity-dependent decrease in ZDHHC9 palmitoylation might be specific to certain types of synaptic stimuli (Fig. 6E,F). Finally, at 40 min post cLTP *in vitro*, we observed a significant reduction in ZDHHC2 phosphorylation (Fig. 6G), along with no changes in ZDHHC2 palmitoylation or total protein levels (Fig. 6G,H). These observations indicate that several of the post-translational changes observed with cLTP *in vitro* are mirrored following a learning event *in vivo*.

### cLTP does not alter APT2 or ABHD17 transcription, protein turnover, post-translational modifications or activity

**Fig. 7.**
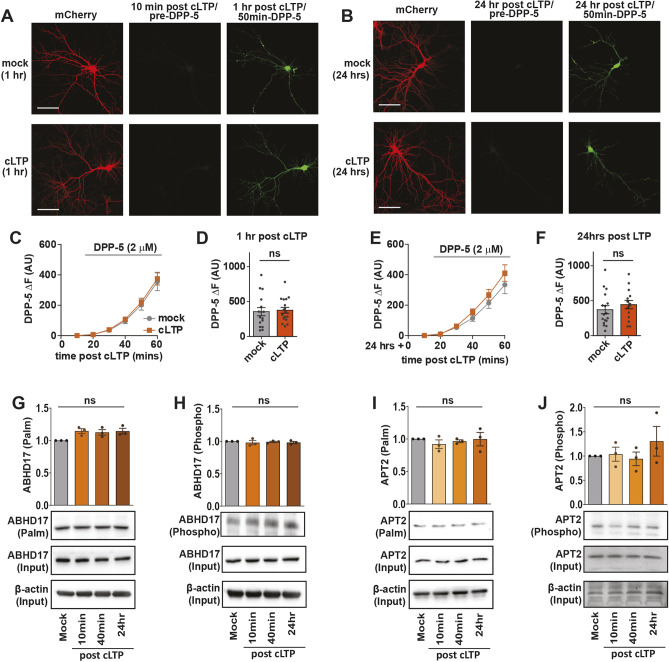
**Depalmitoylating enzyme activity is unchanged following cLTP.** (A) Representative images of the fluorescent depalmitoylation probe-5 (DPP-5; 2 μM) 1 h after cLTP treatment in 14 DIV-cultured hippocampal neurons transfected at 11 DIV with mCherry. Left: mCherry cell fill. Middle: Background fluorescence within mCherry mask prior to addition of DPP-5 to the bath. Right: DPP-5 fluorescence within the mCherry mask 50 mins post addition of DPP-5 to the bath (1 h post cLTP). Scale bars: 100 μm. (B) As A, but 24 h post cLTP treatment. (C) Graph of time-course of DPP-5 fluorescence increase (ΔF) following cLTP and subsequent bath addition of DPP-5. No significant difference was observed between mock- and cLTP-treated neurons at any time point. (D) Graph of DPP-5 fluorescence increase (ΔF) 1 h post cLTP and 50 min post addition of DPP-5 to the bath. No significant difference was observed between mock and cLTP-treated neurons at any time-point. Mock, *n*=16 neurons; cLTP, *n*=17 neurons from two independent cultures. (E,F) As C,D but 24 h post cLTP and 50 min post addition of DPP-5 to the bath. Mock, *n*=17 neurons; cLTP, *n*=14 neurons from three independent cultures. AU, arbitrary units. (G) Acyl-Rac assay showing palmitoylated ABHD17 in cultured hippocampal neurons (14 DIV) following cLTP. Palmitoylated ABHD17 values in the graph were derived from ABHD17 ‘palm’ normalized to ABHD17 ‘input’ and the β-actin loading control. *n=*3 independent hippocampal cultures per condition. (H) Phospho-protein purification assay showing phosphorylated ABHD17 following cLTP. Phosphorylated ABHD17 values in the graph were derived from ABHD17 ‘phospho’ normalized to ABHD17 ‘input’ and the β-actin loading control. *n=*3 independent hippocampal cultures per condition. (I) Acyl-Rac assay showing palmitoylated APT2 in cultured hippocampal neurons (14 DIV) following cLTP. Palmitoylated APT2 values in the graph were derived from APT2 ‘palm’ normalized to APT2 ‘input’ and the β-actin loading control. *n=*3 independent hippocampal cultures per condition. (J) Phospho-protein purification assay showing phosphorylated APT2 following cLTP. Phosphorylated APT2 values in the graph were derived from APT2 ‘phospho’ normalized to APT2 ‘input’ and the β-actin loading control. *n=*3 independent hippocampal cultures per condition. *n=*3 hippocampal cultures per experiment. For all graphs, results are mean±s.e.m. with individual data points shown. ns, not significant (unpaired two-tailed Student's *t*-test for D,F; one-way ANOVA with Tukey's post hoc test for G–J).

Activity-dependent changes in synaptic substrate palmitoylation might also be a result of dynamic regulation of depalmitoylating enzymes. To directly investigate whether synaptic activity impacts depalmitoylating enzyme activity, we used a recently developed probe (depalmitoylation probe-5 or DPP-5) that generates fluorescent signals in response to thioestaerase activity in live cells (Qiu et al., 2018). We first validated that the DPP-5 probe is sensitive enough to detect changes in thioesterase activity in neurons using the pan-thioesterase inhibitor palmostatin B (Fig. S2), and then tested whether thioesterase activity is altered following cLTP induction. We observed no significant changes in DPP-5 fluorescence either 1 h (Fig. 7A,C,D) or 24 h (Fig. 7B,E,F) after cLTP treatment, indicating that there is no change in thioestaerase activity following cLTP. As the membrane-anchored serine hydrolases, ABHD17A, ABHD17B and ABHD17C, have the highest depalmitoylating activity against PSD-95 in neurons (Yokoi et al., 2016), we focused our assays on these enzymes. We observed no activity-induced changes in ABHD17 total protein levels (phosphor-assay input, *P*=0.858, Fig. 7G), palmitoylation (Fig. 7G) or phosphorylation (Fig. 7H) (ABHD17 antibody validation in Fig. S1). Taken together, these data suggest that activity-dependent changes in PSD-95 palmitoylation are mediated through post-translational regulation of ZDHHC2 as opposed to regulation of ABHD17 function. We also assessed post-translational modifications of APT2, which is expressed in hippocampal neurons (Wild et al., 2022), and again found no changes in APT2 protein levels (Fig. 7J; phospo-assay input, *P*=0.753), palmitoylation (Fig. 7I), or phosphorylation (Fig. 7J). Finally, we found no changes in the transcription of any of the depalmitoylating enzymes tested following cLTP (Fig. S3). In summary, we did not find evidence of cLTP induced activity-dependent changes in depalmitoylating enzyme activity or post-translational regulation, indicating that the ZDHHC enzymes might be the primary regulators of synaptic activity-induced differential palmitoylation of substrate proteins.

## DISCUSSION

Although accumulating evidence indicates that the dynamic palmitoylation of many synaptic proteins occurs in response to changes in synaptic activity and is critical for synaptic plasticity, the mechanism by which this occurs is largely unknown. Given the reversible nature of palmitoylation, changes in protein palmitoylation might reflect changes in palmitoylation and/or depalmitoylation rates. Our study set out to elucidate the post-translational modifications regulating the palmitoylation machinery that might ultimately modulate enzyme function and activity-induced palmitoylation of synaptic substrates. We performed a systematic screen of endogenous post-translational modifications for numerous neuronal palmitoylating and depalmitoylating enzymes, as well as an investigation of how these modifications might alter protein function or substrate interactions.

### cLTP induces changes in post-translational modifications of ZDHHC5, but not ZDHHC8

Our previous work demonstrated that glycine cLTP induction results in the transient removal of ZDHHC5 from the synaptic membrane, followed by a 2-fold increase in ZDHHC5 membrane localization 20 mins later (Brigidi et al., 2015). These changes in trafficking were accompanied by increased δ-catenin palmitoylation and AMPAR stabilization at the synapse. Here, we also demonstrate a 2.5-fold decrease in total ZDHHC5 protein levels at the same time point, which is a result of activity-dependent phosphorylation of serine and threonine residues (Ser569, Ser573 and Thr574) within the polo-box domain of the ZDHHC5 C-terminal region. How then might both increased synaptic localization, and overall protein degradation of ZDHHC5 work cooperatively to alter synaptic function? Recruiting ZDHHC5 to the synaptic membrane and degrading non-synaptic ZDHHC5 could profoundly impact the palmitoylation of its downstream substrates that are localized to different compartments. For example, although palmitoylation of δ-catenin stabilizes AMPAR at the synaptic membrane, palmitoylation of the neuronal scaffolding protein GRIP1B by ZDHHC5 enhances AMPAR turnover (Thomas et al., 2012). It is therefore interesting to speculate that enhanced synaptic activity might differentially regulate ZDHHC5 to stabilize AMPARs by simultaneously increasing the palmitoylation of δ-catenin and decreasing the palmitoylation of GRIP1B in different subcellular compartments.

We also observed an increase in ZDHHC5 palmitoylation following cLTP. Several cysteine residues in the C-terminal region of ZDHHC5 are known to be palmitoylated (Cys236, Cys237 and Cys245 in the human isoform), which lie close to a juxtamembrane amphipathic helix. This helix forms part of the binding site for ZDHHC5 accessory protein GOLGA7B (Woodley and Collins, 2019) and the Na^+^-K^+^ ATPase Na^+^ pump (Plain et al., 2020). Furthermore, ZDHHC20 was identified as the enzyme that can palmitoylate these residues in ZDHHC5 in HEK293 cells (Plain et al., 2020). It is therefore possible that activity-dependent palmitoylation of ZDHHC5 could be mediated by ZDHHC20, or alternatively by ZDHHC enzymes that colocalize with ZDHHC5 in neurons that are yet to be identified. Previous work from our group found that mutation of the aforementioned C-terminal ZDHHC5 cysteine residues increased surface localization of ZDHHC5 in rat hippocampal neurons (Shimell et al., 2019), raising the possibility that activity-dependent increases in ZDHHC5 palmitoylation could be another means to facilitate ZDHHC5 endocytosis and re-localization.

Surprisingly, we did not observe any activity-dependent post-translational modifications for ZDHHC8, an enzyme that shares a high degree of sequence homology with ZDHHC5 (60%), and that might therefore be expected to be responsive to neuronal activity. ZDHHC5 contains many regulatory motifs that are also found in ZDHHC8, including a C-terminal PDZ-binding domain (Thomas et al., 2012), tyrosine endocytic motif (Brigidi et al., 2015) and a polo-box like domain (ZDHHC8 putative polo-box sequence: DSGVYDT), which can be phosphorylated at Ser573 and Thr578 according to PhosphoSite (https://www.phosphosite.org/). It is therefore possible that phospho-regulation of the ZDHHC8 polo box could be induced by stimuli other than cLTP*.* Additionally, ZDHHC5 and ZDHHC8 are functionally redundant in palmitoylating common substrates such as ankyrin-G (ANK3) in polarized epithelial cells (He et al., 2014). However, the two enzymes show distinct dendritic subcellular localizations in neurons (Thomas et al., 2012), and analysis of *Zdhhc5* and *Zdhhc8* expression in the mouse brain revealed that they are differentially enriched within regional neuronal subpopulations of the hippocampus and cortex (Wild et al., 2022). Our findings here that ZDHHC5 and ZDHHC8 are differentially responsive to synaptic activity in the hippocampus support the notion that these two enzymes might also have distinct roles in regulating neuronal function and synaptic plasticity.

Previous studies have reported activity-dependent phospho-regulation of ZDHHC5 and ZDHHC8, including a recent study that revealed that phosphorylation at Tyr91 within the intracellular catalytic loop by LYN kinase in adipocytes reduces ZDHHC5 enzymatic activity (Hao et al., 2020). However, it is not yet clear whether this modification occurs in neurons or whether it is dynamically regulated by neuronal activity. A recent phospho-proteomic screen identified several serine residues in rat ZDHHC5 that were differentially phosphorylated following changes in neuronal activity, including Ser380, Ser432 and Ser621, as well as ten other serine/threonine phosphosites that were not altered by activity (Desch et al., 2021). Interestingly, aside from ZDHHC5, the only other ZDHHC that was identified to be differentially phosphorylated following activity changes in neurons was at a single residue in ZDHHC8 (Ser335; Desch et al., 2021). A previous study also found that BDNF/TrkB signaling can stimulate PKMζ phosphorylation of ZDHHC8 in cortical neurons, indicating that activity-dependent phospho-regulation of ZDHHC8 is possible during different types of neuronal activity (Yoshii et al., 2011). Overall, we have found in this study that numerous activity-dependent post-translational modifications regulate ZDHHC5, supporting previous studies that have identified ZDHHC5 as a key mediator of dynamic synaptic substrate palmitoylation following changes in neuronal activity.

### ZDHHC9 palmitoylation is decreased following cLTP

We found that cLTP in can induce a decrease in palmitoylation within the ZDHHC9 catalytic DHHC domain. This is accompanied by a decrease in the palmitoylation of known ZDHHC9 substrates TC10 and N-RAS (Shimell et al., 2019), indicating a potential role for activity-regulated control of ZDHHC9 enzymatic activity. Previous studies from our group revealed that knock-down or knock-out of ZDHHC9 reduces the palmitoylation of TC10 and N-RAS (Shimell et al., 2019), supporting the notion that decreased substrate palmitoylation might be a direct result of reduced ZDHHC9 function. It is unclear how neuronal activity might lead to decreased ZDHHC9 palmitoylation. It is possible that cLTP could alter ZDHHC9 accessibility to palmitoylating or depalmitoylating enzymes. As the decreased palmitoylation of ZDHHC9 appears to occur within the catalytic domain of the protein, it is possible that cLTP could alter the interaction of ZDHHC9 with its cofactor, GOLGA7, leading to a decrease in ZDHHC9 autopalmitoylation. However, we did not observe a concurrent decrease in ZDHHC9 total protein levels, which might be expected if the interaction was disrupted (Swarthout et al., 2005), indicating that a mechanism other than a decrease in GOLGA7 binding might be responsible for decreased ZDHHC9 palmitoylation. Other mechanisms that alter ZDHHC9 autopalmitoylation within the active site might also be responsible.

Our observation of activity-dependent regulation of ZDHHC9 is particularly interesting, given the important role that this enzyme plays in neuronal outgrowth and synapse formation, two processes that are impaired when the catalytic cysteine residue of ZDHHC9 is mutated to serine (Shimell et al., 2019). Future work is needed to determine the functional consequences of activity-dependent regulation of ZDHHC9 palmitoylation.

### ZDHHC2 phospho-regulation alters substrate interactions

In this study, we observed an increase in PSD-95 palmitoylation following cLTP as previously reported (Brigidi et al., 2015; Nasseri et al., 2022). However, we also observed a reduction in ZDHHC2/PSD-95 interactions following cLTP, which might initially appear counterintuitive. One possible explanation is that changes in the phosphorylation of ZDHHC2 following cLTP impacts ZDHHC2 enzyme kinetics, resulting in a more rapid transfer of palmitic acid to substrate proteins and, hence, a decrease in the duration of interaction with substrates. It is also possible that changes in ZDHHC2 phosphorylation changes the site(s) of ZDHHC2/PSD-95 interaction, resulting in less interaction but greater palmitoylation. Compensation by other ZDHHC enzymes could explain our result; however, previous work has found PSD-95 to be a substrate for a limited number of ZDHHC enzymes including ZDHHC2, ZDHHC3, ZDHHC7 and ZDHHC15, with only ZDHHC2 and ZDHHC15 participating in activity-dependent PSD-95 palmitoylation (Noritake et al., 2009). However, given that the expression of ZDHHC15 is very low in the hippocampus, ZDHHC2 is thought to be the primary palmitoylating enzyme for PSD-95 (Wild et al., 2022; Noritake et al., 2009). Although further work is required to fully explain the mechanism, it is likely that ZDHHC2 dephosphorylation mediates the increase in PSD-95 palmitoylation.

Although the binding site of PSD-95 on ZDHHC2 has not been precisely mapped, the mutated serine and threonine residues in the HA–ZDHHC2-8×A phospho-dead mutant are unlikely to cause structural changes in the protein that might disrupt PSD-95 binding, as these residues are located within the distal C-terminal region (330–366 aa), which is predicted to be predominantly unstructured (determined using IUPred3; https://iupred3.elte.hu/). It will be necessary in the future to identify the kinases and phosphatases that modify ZDHHC2. Using the prediction software KinasePhos3 (Ma et al., 2022), we found that the ZDHHC2 C-terminal region has a very strong probability of being a substrate for a number of kinases, including PKD1, AMPK1, CAMK2A and PKA. Notably, both CAMK2A and PKA are known to be highly responsive to LTP stimuli, indicating that these enzymes might play a role in dynamic phosphorylation of ZDHHC2 (Woolfrey and Dell'Acqua, 2015).

### *In vivo* changes in ZDHHC post-translational modifications

Many of the ZDHHC post-translational changes we observed following cLTP *in vitro* were also replicated following cFC *in vivo*, including a striking decrease in both ZDHHC5 total protein levels and ZDHHC2 phosphorylation. Recent work has revealed that relatively few neurons and synapses in the hippocampus undergo consolidative plasticity to become so-called ‘engram’ neurons following learning stimuli such as cFC (Denny et al., 2014; Josselyn and Tonegawa, 2020; Liu et al., 2012). However, it has also been reported that in addition to cell-autonomous mechanisms regulating engram formation, numerous non-engram cells are activated following cFC, including non-engram excitatory neurons, inhibitory neurons, neural progenitors and glia (Denny et al., 2014; Li et al., 2020; Pan et al., 2020; Seo et al., 2015; Stefanelli et al., 2016). Given the magnitude of the changes we have observed here, our results would indicate that ZDHHC post-translational changes extend beyond the relatively small population of engram neurons, to the wider network of hippocampal cells that are activated following cFC that support engram formation. Furthermore, our findings support the notion that multiple types of synaptic stimuli can drive similar changes in ZDHHC post-translational modifications.

### Evidence for activity-dependent regulation of palmitoylating, but not depalmitoylating, enzymes

Despite reports of both activity-dependent increases and decreases in palmitoylation in the hippocampus (Nasseri et al., 2022), evidence of activity-dependent regulation of the family of depalmitoylating enzymes is currently lacking. The findings in this study are consistent with a recent phospho-proteomic screen that did not report activity-dependent changes in the phosphorylation of any of the best-characterized depalmitoylating enzymes, including APT1, APT2, PPT1 and all members of the ABHD17 family (Desch et al., 2021). It is possible that more detailed future studies might reveal novel mechanisms that regulate the family of depalmitoylating enzymes and promote activity-dependent changes in substrate palmitoylation. However, given the current evidence, we propose that the ZDHHC family of proteins are the primary sensors of neuronal activity, and that their bidirectional regulation is the effector of both increases and decreases in neuronal substrate palmitoylation.

## MATERIALS AND METHODS

### DNA constructs and primers

Plasmids encoding N-terminal HA-tagged mouse ZDHHC1–9 and ZDHHC11–24, HA-tagged ZDHHC5-AAA, HA-tagged P35 and myc-tagged CDK5 were kind gifts from Dr Gareth M. Thomas (Temple University, Philadelphia, PA, USA). Plasmids encoding FLAG-tagged ABHD17A, -ABHD17B and -ABHD17C were kind gifts from Dr Elizabeth Conibear (University of British Columbia, Vancouver, BC, Canada). Plasmids encoding myc-tagged PLK2 and myc-tagged PLK2 kinase-dead mutant were kind gifts from Dr Daniel Pak (Georgetown University, Washington, DC, USA). shRNA against ZDHHC5 was a kind gift from Dr Richard Huganir (Johns Hopkins University, Baltimore, MD, USA). For qRT-PCR, the primers used are listed in Table S1.

### Antibodies

#### Primary antibodies

The following primary antibodies were used: anti-β-actin (1:5000, Sigma-Aldrich, A1978), anti-ZDHHC1 (1:1000, Abcam, ab223042), anti-ZDHHC2 (1:1000, Santa Cruz Biotechnology, sc-515204), anti-ZDHHC2 (1:500, Sigma-Aldrich, SAB1101457), anti-ZDHHC3 (1:500, Aviva Systems Biology, ARP59576), anti-ZDHHC3 (1:500, Sigma-Aldrich, SAB2107413), anti-ZDHHC3 (1:1000, Abcam, ab124084), anti-ZDHHC3 (1:1000, Abcam, ab31837), anti-ZDHHC4 (1:500, Aviva Systems Biology, ARP78440), anti-ZDHHC5 (1:1000 for western blotting, 5 µg for immunoprecipitation, Sigma-Aldrich, HPA014670), anti-ZDHHC6 (1:600, Abcam, ab121423), anti-ZDHHC7 (1:500, Aviva Systems Biology, OAAB11570), anti-ZDHHC7 (1:500, Boster Bio, A11785), anti-ZDHHC8 (1:500, Santa Cruz Biotechnology, sc-374191), anti-ZDHHC9 (1:1000, Sigma-Aldrich, SAB4502104), anti-ZDHHC9 (1:1000, Thermo Fisher Scientific, PA5-26721), anti-ZDHHC11 (1:500, Abcam, ab116065), anti-ZDHHC12 (1:500, Aviva Systems Biology, ARP60674), anti-ZDHHC13 (1:500, Aviva Systems Biology, ARP44398), anti-ZDHHC14 (1:500, Aviva Systems Biology, ARP42628), anti-ZDHHC15 (1:500, Sigma-Aldrich, SAB4500608), anti-ZDHHC15 (1:200, Abcam, ab121203), anti-ZDHHC15 (1:500, Santa Cruz Biotechnology, sc-169847), anti-ZDHHC15 (1:500, Thermo Fisher Scientific, PA5-39327), anti-ZDHHC16 (1:500, Aviva Systems Biology, ARP50063), anti-ZDHHC17 (1:300, Proteintech 15465-1-AP), anti-ZDHHC17 (1:500, Sigma-Aldrich, AV47141), anti-ZDHHC18 (1:1000, Abcam, ab154790), anti-ZDHHC19 (1:500, Abcam, ab179545), anti-ZDHHC20 (1:500, Aviva Systems Biology, ARP72069), anti-ZDHHC21 (1:300, Abcam, ab103755), anti-ZDHHC22 (1:500, Santa Cruz Biotechnology, sc-514005), anti-phospho-PLK binding motif (ST*P) (1:1000, Cell Signaling Technology, 5243S), anti-HA (1:1000, Cell Signaling Technology, C29F4), anti-myc (1:1000, Cell Signaling Technology, 2276), anti-GFP (1:3000, Abcam, ab290), anti-ABHD17 (1:1000, Origene TA331704), anti-ABHD17 (1:1000, Proteintech, 15854-1-AP), anti-FLAG (1:1000, Sigma-Aldrich, F7425), anti-PSD-95 (1:500, Abcam, ab2723), anti-APT2 (1:500, Abcam, ab151578), anti-TC10 (1:1000, Abcam, ab168645) and anti-N-Ras (1:500, Santa Cruz Biotechnology, sc-31).

#### Secondary antibodies

The following secondary antibodies were used: goat anti-mouse IgG-HRP (1:6000, Bio-Rad, 170-6516) and goat anti-rabbit IgG-HRP (1:6000, Bio-Rad, 170-6515).

### Cell culture

#### Primary hippocampal neurons

All procedures involving animals were approved by the Canadian Council of Animal Care and the University of British Columbia Committee on Animal Care. Hippocampi from embryonic day 18 Sprague Dawley rats of either sex were prepared as previously described (Xie et al., 2000). Briefly, hippocampi were dissected and incubated with 0.25% trypsin (Thermo Fisher Scientific) and 0.05% DNase (Thermo Fisher Scientific) for 20 and 3 mins, respectively. Cells were dissociated with titration and plated at a density of 3.2 million per 10-cm culture dish for biochemical assays. Cells were allowed to adhere in plating medium containing minimum essential medium (MEM; Gibco, Thermo Fisher Scientific), supplemented with 10% (vol/vol) heat-inactivated-fetal bovine serum (FBS) (Gibco, Thermo Fisher Scientific), sodium pyruvate (Gibco, Thermo Fisher Scientific), 0.5% glucose, GlutaMAX (Gibco, Thermo Fisher Scientific) and penicillin/streptomycin (Gibco, Thermo Fisher Scientific). After 3 h, the plating medium was replaced with maintenance medium containing neurobasal medium (Gibco, Thermo Fisher Scientific) supplemented with NeuroCult SM1 (StemCell, instead of B27 in the original protocol), GlutaMAX and penicillin/streptomycin. Cultures were maintained at 37°C and 5% CO_2_.

#### HEK293T cells

HEK293T cells (American Type Culture Collection; CRL-1573; authenticated by STR profiling and confirmed negative for mycoplasma) were aliquoted into a 10 cm culture dish with 15 ml of pre-warmed (37°C) Dulbecco's modified Eagle medium (Gibco, Thermo Fisher Scientific), supplemented with 10% (vol/vol) heat-inactivated FBS and 1% penicillin/streptomycin. HEK293T cells were maintained in an incubator at 37°C and 5% CO_2_.

### Transfection

#### Primary hippocampal cultures – transient transfections

Neurons were transfected at 9–11 days *in vitro* (DIV) using Lipofectamine 2000 (Invitrogen) according to the manufacturer's protocol and used for experiments on DIV 12–15.

#### Primary hippocampal cultures – Amaxa nucleofection

Neurons were nucleofected with the identified plasmids prior to plating at 0 DIV using Amaxa Rat Neuron Nucleofector kit (DGP-1003; Lonza) according to the manufacturer's protocol. Cells were then used for experiment at 13–15 DIV.

#### HEK293T cells

HEK293T cells were transfected at 70–80% confluency using Lipofectamine 2000 (Invitrogen) according to the manufacturer's recommendations and used for experiments 24–48 h after transfection.

### Neuronal stimulation (cLTP)

Neuronal activity was enhanced as per the previously published protocol (Lu et al., 2001). Briefly, at 13–15 DIV, the maintenance medium was removed and stored at 37°C and cells were washed three times with pre-warmed (37°C) Mg^2+^-free extracellular solution made of 140 mM NaCl, 1.3 mM CaCl_2_, 5.0 mM KCl, 25 mM HEPES and 33 mM glucose, supplemented with 0.0005 mM tetrodotoxin (TTX-citrate, Tocris) and 0.001 mM strychnine (Sigma-Aldrich) (pH 7.4). To chemically induce LTP, cells were incubated with the above extracellular solution supplemented with 200 µM glycine for 3 min. Cells were then washed two times with the above extracellular solution containing 2 mM MgCl_2_, and then replaced with the stored maintenance medium. Neuronal cells were maintained in a 37°C incubator with 5% CO_2_ for the indicated time prior to experimentation. Control cells were treated the same as experimental groups but were not exposed to glycine during the 3-min incubation.

### RNA isolation and qRT-PCR

At 15 DIV, hippocampal cultured neurons were stimulated as described above and mRNA was isolated after identified time points using TRIzol Reagent (Thermo Fisher Scientific) according to the manufacturer's instructions. Approximately 200 ng of total DNA-free RNA was reverse transcribed using Verso cDNA Synthesis Kit (Thermo Fisher Scientific). The cDNA was then quantified by qPCR using SYBR green (Thermo Fisher Scientific). qRT-PCR analysis was performed at the Biomedical Research Center at the University of British Columbia using a 7900HT Real-Time PCR thermocycler machine (Applied Biosystems). mRNA levels of genes of interest were normalized to *Gapdh* and shown as fold change over baseline using the ΔΔCT method (Schmittgen and Livak, 2008).

### Western blot assay

Brain tissue, primary hippocampal neurons and HEK293T cells were washed with ice-cold PBS and lysed in ice-cold Tris lysis buffer containing 1% IGEPAL (Sigma-Aldrich), 50mM Tris-HCl pH 7.5, 150mM NaCl and 10% glycerol, supplemented with phenylmethanesulfonyl fluoride (PMSF) solution and a protease inhibitor cocktail with EDTA (Roche). The samples were vortexed, run through a 26-gauge syringe and kept at 4°C to nutate for 30 min. Lysates were then cleared by spinning down at 16,000 ***g*** for 30 min at 4°C. Protein quantification was done using a BCA assay kit (Thermo Fisher Scientific) as per the manufacturer's instructions. Proteins were separated by electrophoresis on a 10–12% SDS-PAGE gel. Proteins were then transferred to a PVDF membrane (Bio-Rad) and blocked for 1 h in 3–5% bovine serum albumin in Tris-buffered saline containing 0.1% Tween (TBST). The membrane was then incubated overnight at 4°C with the identified primary antibody. The membranes were then washed three times for 15 min in TBST at room temperature with agitation and incubated with the appropriate secondary antibodies for 1 h at room temperature, before being washed three times for 15 min with TBST. Proteins were visualized using chemiluminescence (Immobilon Western Chemiluminescent HRP Substrate, Millipore, WBKLS0500) on a Bio-Rad ChemiDoc (XRS**+**). Blots were quantified using ImageJ software. For reprobing, blots were stripped as per a previously published protocol (Yeung and Stanley, 2009). For western blot analysis, the input band for the protein of interest was first normalized to β-actin as a loading control. For Acyl-Rac palmitoylation and phospho-protein assays, the amount of palmitoylated or phosphorylated protein was then normalized to the β-actin normalized input. Note that some blots were cut, stripped and/or reprobed as detailed in Fig. S4, and several loading controls are duplicated where they apply to more than one figure panel.

### Immunoprecipitation

Cells were lysed as described above and incubated overnight at 4°C with antibodies under gentle rotation. Then, 80–100 μl of a mix of protein A- and protein G-Agarose beads (Roche) was added to the samples, the beads were recovered 4 h later and then washed five times with ice-cold Tris lysis buffer. Proteins were eluted from beads by heating in 2× SDS loading buffer for 5 min at 80°C. Samples were analyzed by SDS-PAGE, then immunoblotted with the identified antibodies.

### Acyl-Rac assay

Protein palmitoylation assay was performed using CAPTUREome S-palmitoylated protein kit (Badrilla, Leeds, UK), according to the manufacturer's protocol, with the following modification: the protein concentration was measured after dissolving the precipitated protein, to ensure starting with equal protein concentrations. Hippocampal tissue or cultured neurons were lysed and incubated with blocking reagent to block all free thiol groups. The extracted proteins were then acetone precipitated. The pellets were re-dissolved and the protein concentration was measured using BCA assay. The palmitate groups on proteins were cleaved using the thioester cleavage reagent. Proteins with newly liberated thiols were then captured using CAPTUREome resin. The captured proteins were then eluted from the resin. The samples were analyzed by SDS-PAGE, then immunoblotted with the identified antibodies.

### Phospho-protein purification assay

Protein phosphorylation assay was performed using the PhosphoProtein Purification Kit (QIAGEN), exactly as per the guidelines described by the manufacturer. For the negative control, the lysates were incubated with 800 units of λ protein phosphatase (New England Biologicals) for 45 mins at room temperature.

### Context-dependent fear conditioning

Nine-week-old male mice were first habituated by handling 15 min per day for 3 days. On the training day, mice were placed in the conditioning chamber designed by CleverSys with a shock floor and habituated for 2 min. The conditioned group then received a 0.3 mA foot shock for 5 s, whereas the control group did not. One hour later, mice were placed back into the same conditioning chamber for 5 min, and the total freezing time was quantified using FreezeScan software by CleverSys. Immediately after testing, the mice were euthanized and hippocampi isolated and used in Acyl-Rac and phospho-protein purification assays for further palmitoylation and phosphorylation analysis.

### Live-cell imaging of thioesterase activity with DPP-5

Cultured hippocampal neurons were transfected at 12–13 DIV with mCherry (0.8 μg). For experiments with palmostatin B (Sigma-Aldrich), either control (DMSO, 1:1000) or palmostatin B (5 μM; from 5 mM stock in DMSO) solutions were added to the culture medium 30 mins prior to the experiment and were included throughout the imaging experiment. Mock or cLTP treatment was performed as described above. At the time points specified following treatment, neurons were transferred to an imaging chamber at 20°C with an artificial cerebrospinal fluid medium containing 135 mM NaCl, 5 mM KCl, 25 mM HEPES, 10 mM glucose, 2 mM CaCl_2_ and 1 mM MgCl_2_ at pH 7.4. Time-lapse images were acquired using a Zeiss LSM 880 AxioObserver Airyscan microscope with a 20× air objective using a 568 nm laser (mCherry) or a 488 nm laser (DPP-5) with AiryScan Fast mode. Two-color *z*-stack images of the soma and dendritic arbor covering a 422.0×422.0 μm field of view were acquired every 10 mins for a total of 50 mins. After the first acquisition in the time-lapse series to measure background green fluorescence, DPP-5 (2 μM; prepared in-house by the lab of B.C.D.; Qiu et al., 2018) was added to the imaging chamber. Images were maximum-intensity projected prior to analysis. To measure the amplitude of DPP-5 fluorescence changes, a binary mask was drawn of the soma and dendrites from the mCherry cell-fill that was then converted into a region of interest to measure DPP-5 fluorescence changes. Data are reported as the change in DPP-5 fluorescence signal in the background-subtracted mask (ΔF) in arbitrary units (AU).

### Statistical analysis

No statistical methods were used to predetermine sample size. The sample size for western blotting and imaging experiments were based on current standards accepted in the field to assess statistical significance. No randomization was performed. Researchers performing experiments and analysis were not blinded to the experimental groups. No data exclusion was performed. All data values are expressed as mean±s.e.m. Unless otherwise noted, statistical analysis was done using unpaired two-tailed Student's *t*-test and one-way ANOVA (with Dunnett's multiple comparisons or Tukey's multiple comparisons) where applicable and defined when *P*<0.05. In all figures, *P<0.05, **P<0.01, ***P<0.001 and ****P<0.0001. All statistical analysis was performed in GraphPad Prism (La Jolla, CA, USA). Figures were generated using Adobe Illustrator CS6 software (Adobe Systems, San Jose, CA).

## Supplementary Material

10.1242/joces.260629_sup1 Supplementary information
