# Supplementary material for: Activity-dependent post-translational regulation of palmitoylating and depalmitoylating enzymes in the hippocampus
Source: J Cell Sci. 2023 Apr 11;136(7):jcs260629. doi: 10.1242/jcs.260629 (PMC10113885; doi:10.1242/jcs.260629)
Supplement: Supplementary information [file joces-136-260629-s1.pdf]

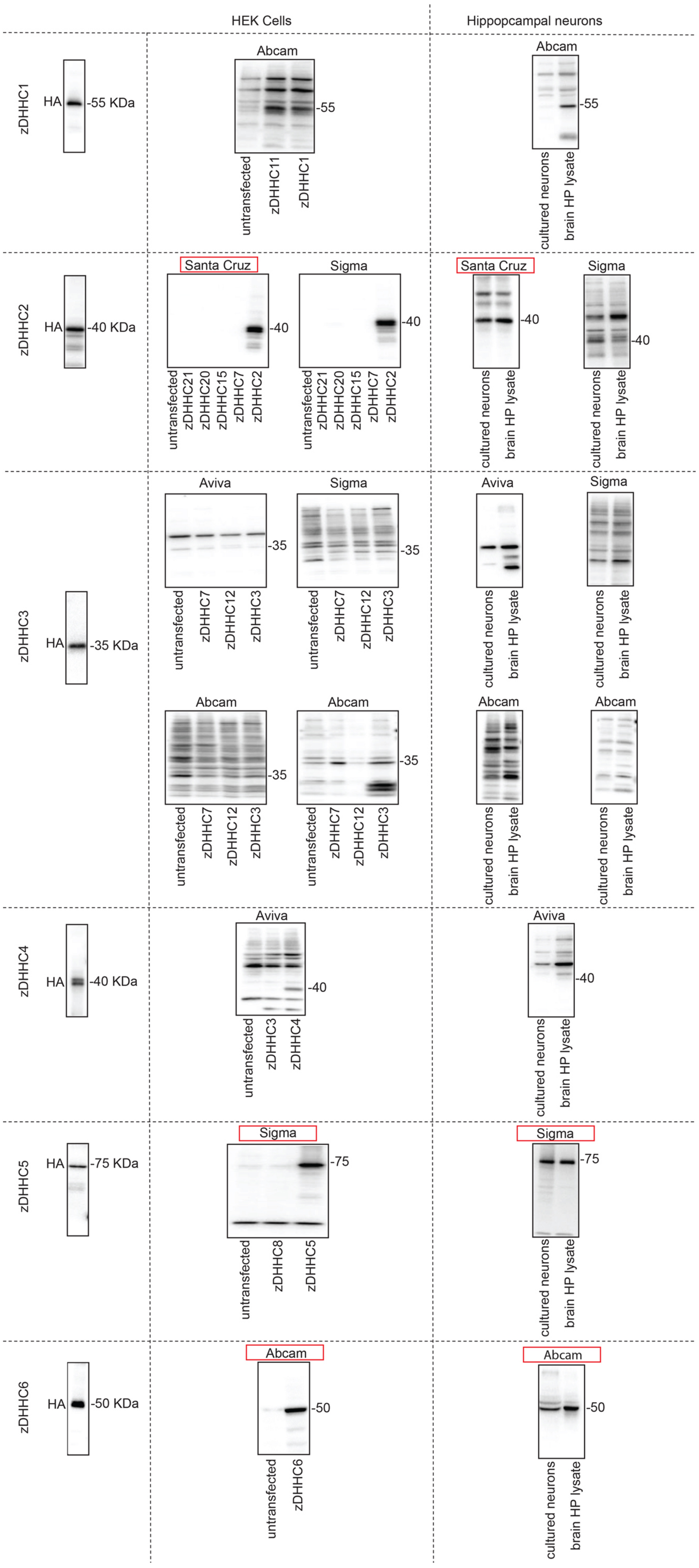

Journal of Cell Science • Supplementary information

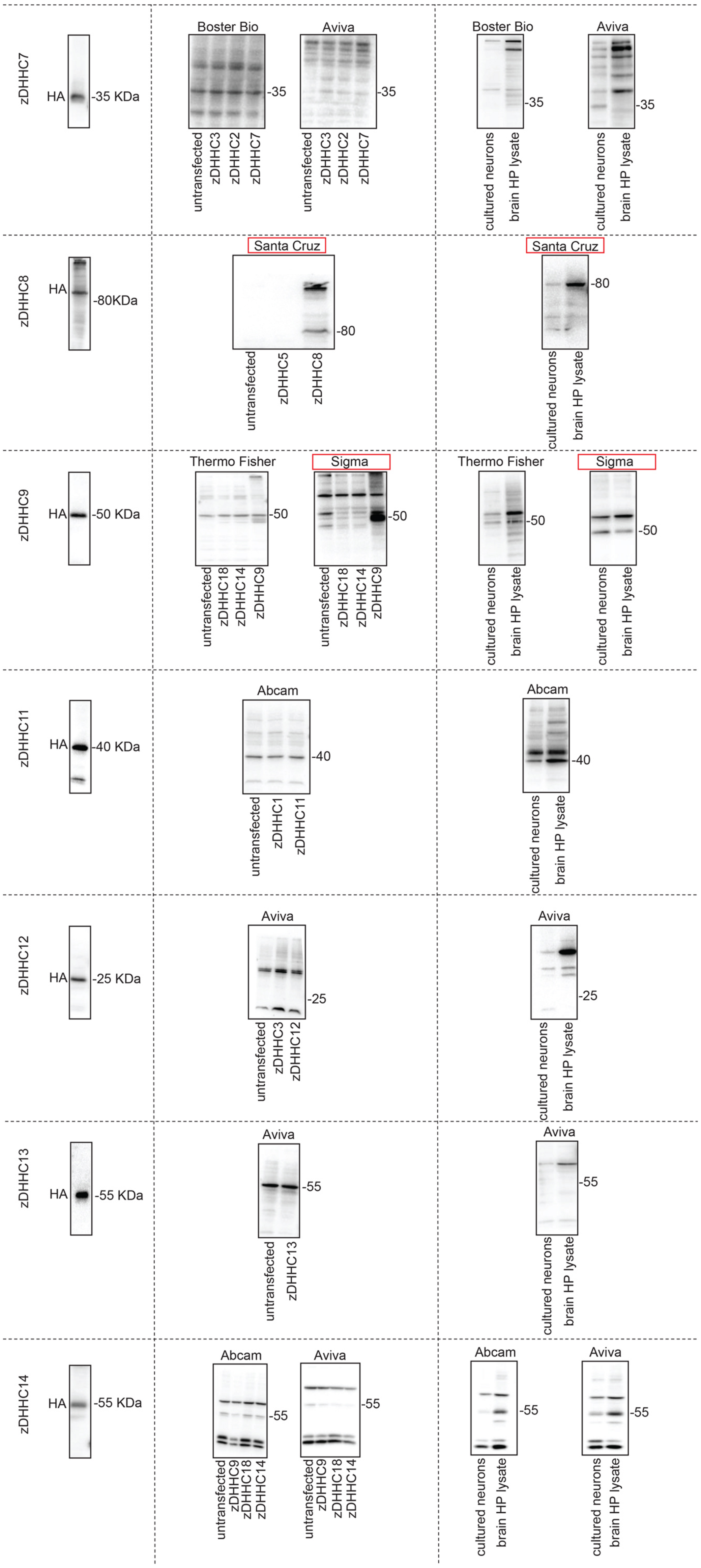

Journal of Cell Science • Supplementary information

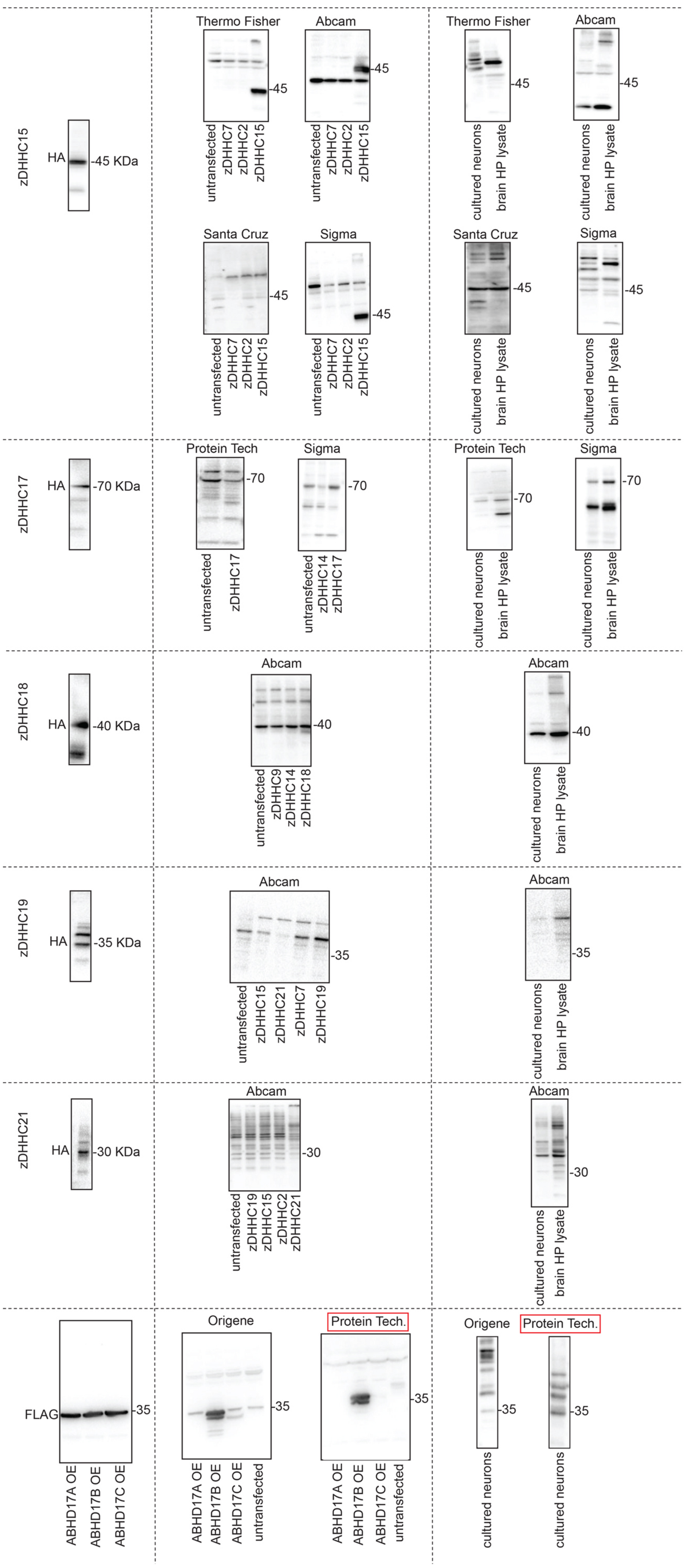

Journal of Cell Science • Supplementary information

Fig. S1. Validation of commercially available zDHHC antibodies

Representative images of western blots testing efficacy and specificity of zDHHC and ABHD17 antibodies. Almost all commercially available antibodies for these enzymes were obtained, and the specificity of each antibody was tested. To do this, each zDHHC enzyme tagged with an HA epitope was transfected into HEK 293T cells. The closest phylogenetic or structural zDHHC for each enzyme was also transfected into HEK 293T cells in parallel. The efficacy and specificity of each antibody were then tested using western blotting. We also used Untransfected HEK cells as a negative control. We then tested the antibodies against endogenous proteins in lysates from either rat cultured hippocampal neurons or rat hippocampus

Left: HEK cells expressing the indicated tagged zDHHC or ABHD and probed for the tag to demonstrate protein expression. Middle: HEK cells expressing the indicated tagged zDHHC and probed with an antibody from the indicated company. Right: Ability of the antibodies to detect endogenous zDHHCs or ABHDs in rat primary hippocampal cultures or hippocampal lysates. Transfection of zDHHC16, 20, and 23 was unsuccessful. Among all tested antibodies only five (marked with red boxes) were shown to be specific for the target proteins.

Journal of Cell Science • Supplementary information

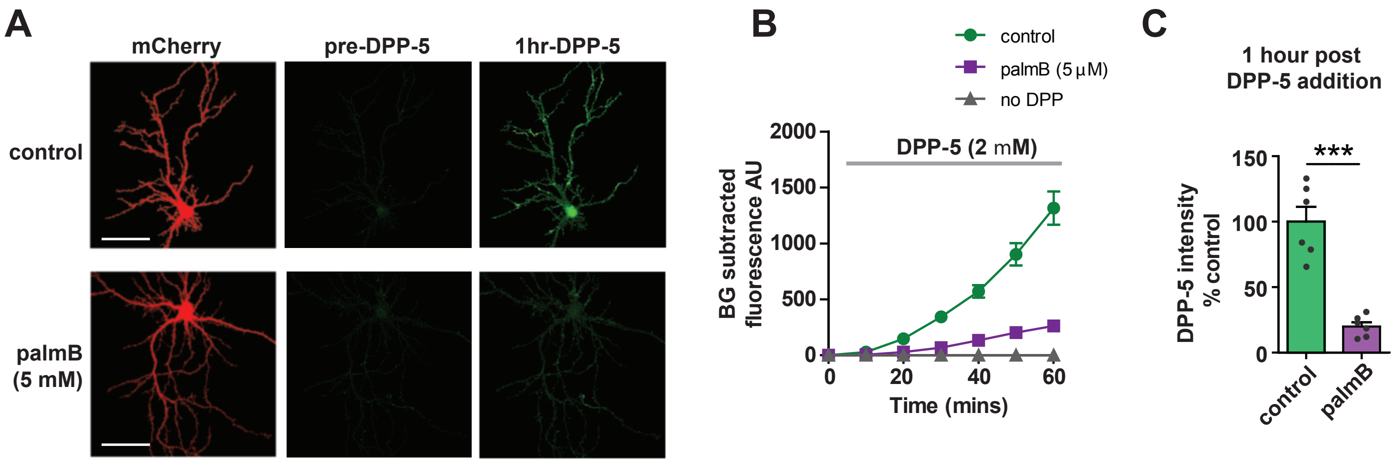

Fig. S2. DPP-5 measures thioesterase activity in cultured hippocampal neurons.

(A) Representative live-cell confocal images of DIV 14 cultured hippocampal neurons before and 1 hour after addition of DPP-5 (2 mM) +/- thioesterase inhibitor palmostatin B (PalmB, 5 μM). Left: mCherry cell fill. Middle: DPP-5 fluorescence in mCherry soma and dendrite mask pre-addition of DPP-5 (2 mM) to imaging chamber. Right: DPP-5 fluorescence in mask 1 hr post DPP-5 addition. Pan-thioesterase inhibitor PalmB substantially decreases DPP-5 fluorescence. Scale bar = 100 mm

(B) Graph showing green flourescence within mCherry cell fill mask after addition of DPP-5 (2 mM) to the bath +/- PalmB. Triangles indicate background fluorescence timecourse without addition of DPP-5.

(C) Graph showing percent inhibition of DPP-5 fluorescence by PalmB.

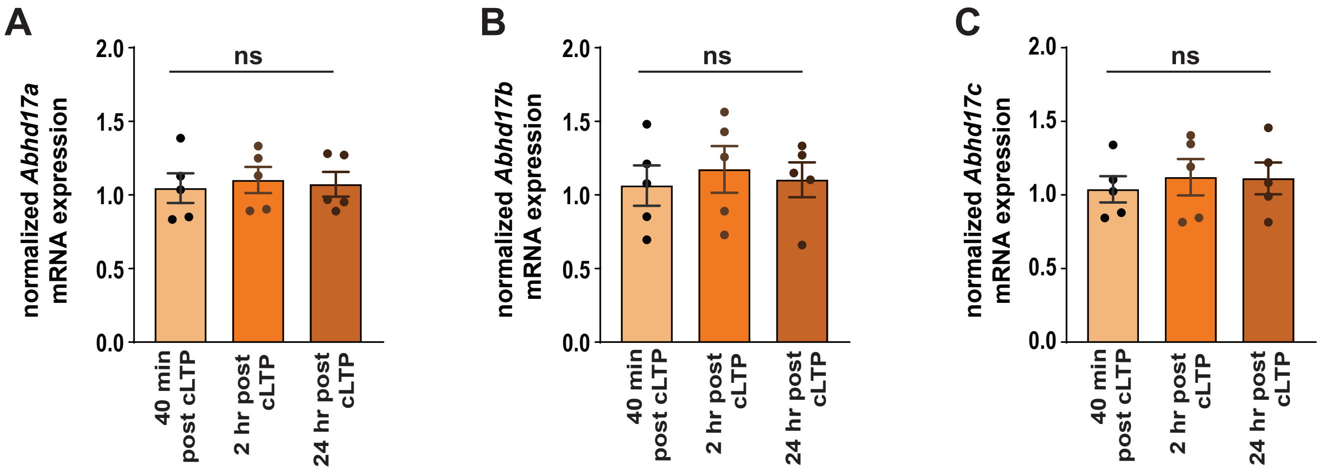

Fig. S3 - qRT-PCR of Abhd17a, b and c following cLTP.

(A) Graph of qRT-PCR data from hippocampal culture lysates showing no change in *Abhd17a* mRNA expression following cLTP treatment at any timepoint. Data points are normalized to mock treated.

(B) As (A) but for *Abhd17b.*

(C) As (A) but for *Abhd17c.*

Journal of Cell Science • Supplementary information

Fig. S4. Blot transparency

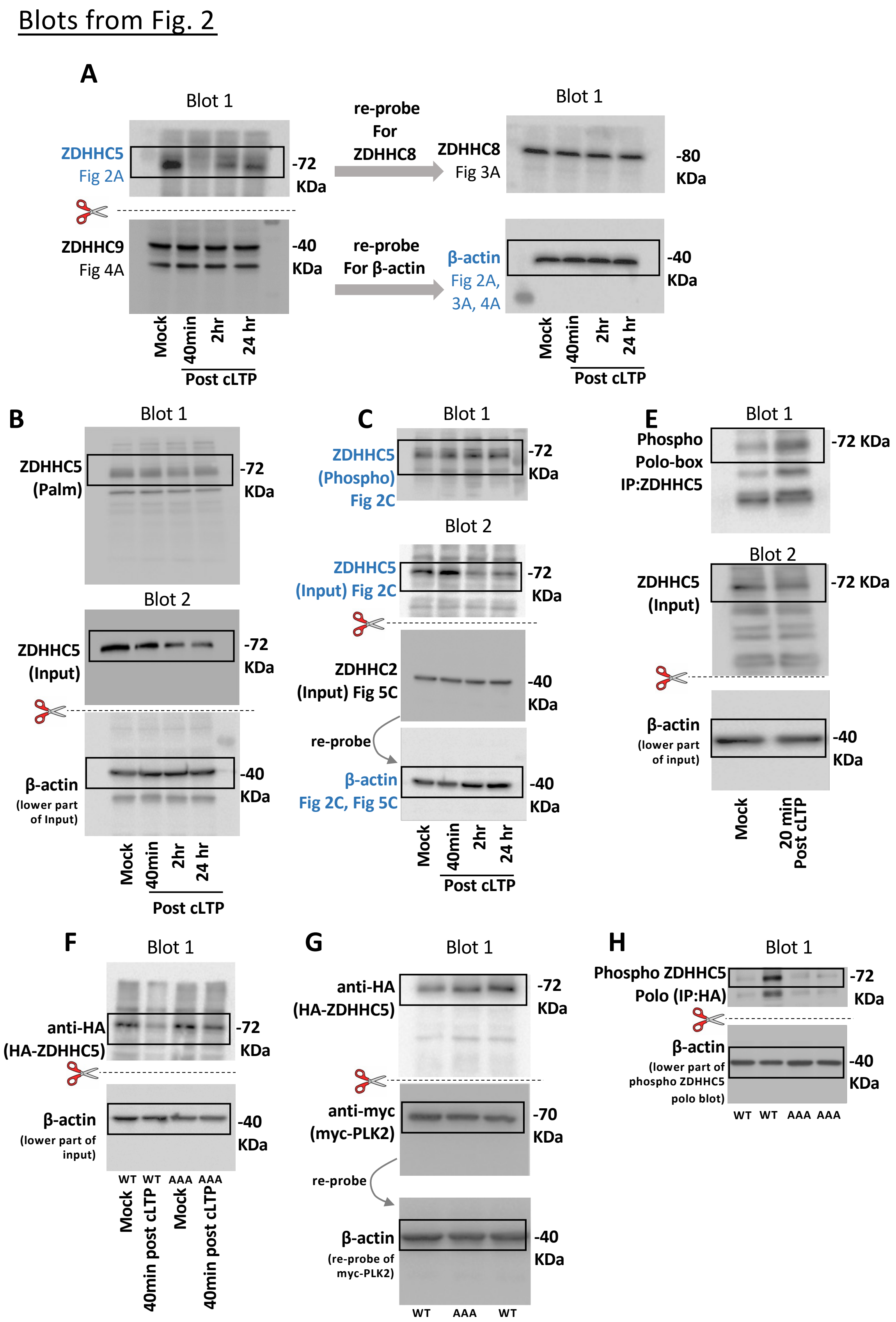

Journal of Cell Science • Supplementary information

Blots from Fig. 2

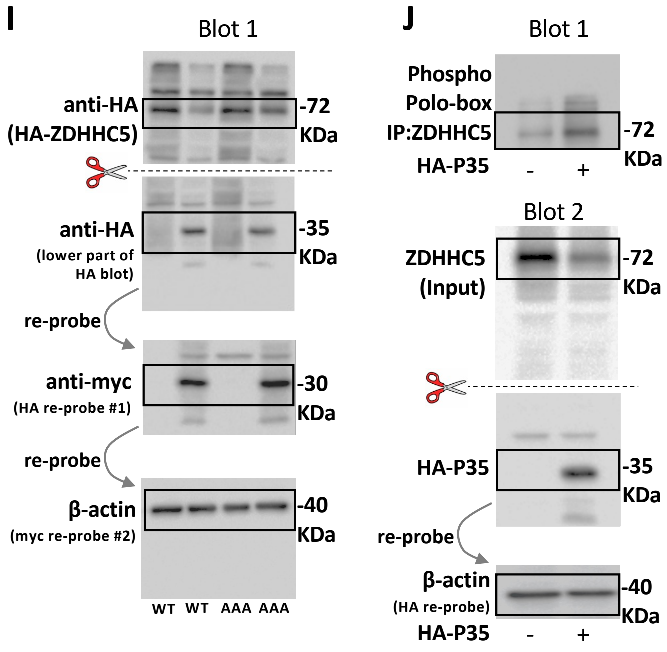

Journal of Cell Science • Supplementary information

Blots from Fig. 3

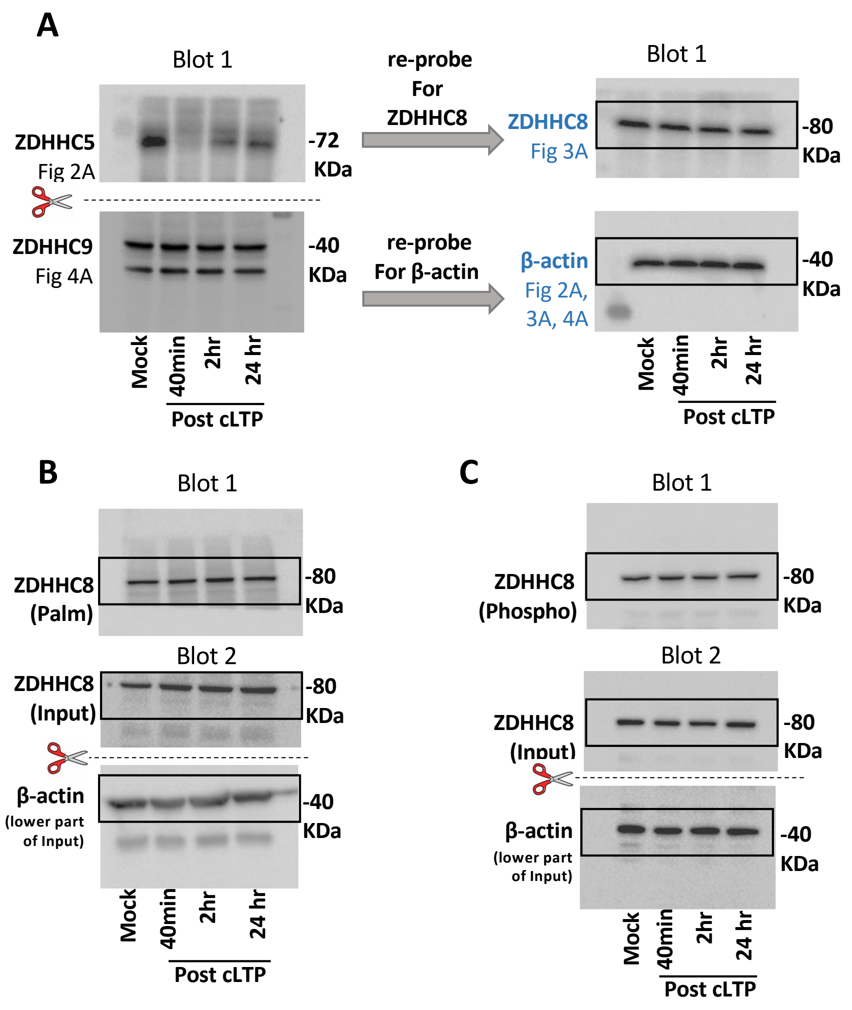

Journal of Cell Science • Supplementary information

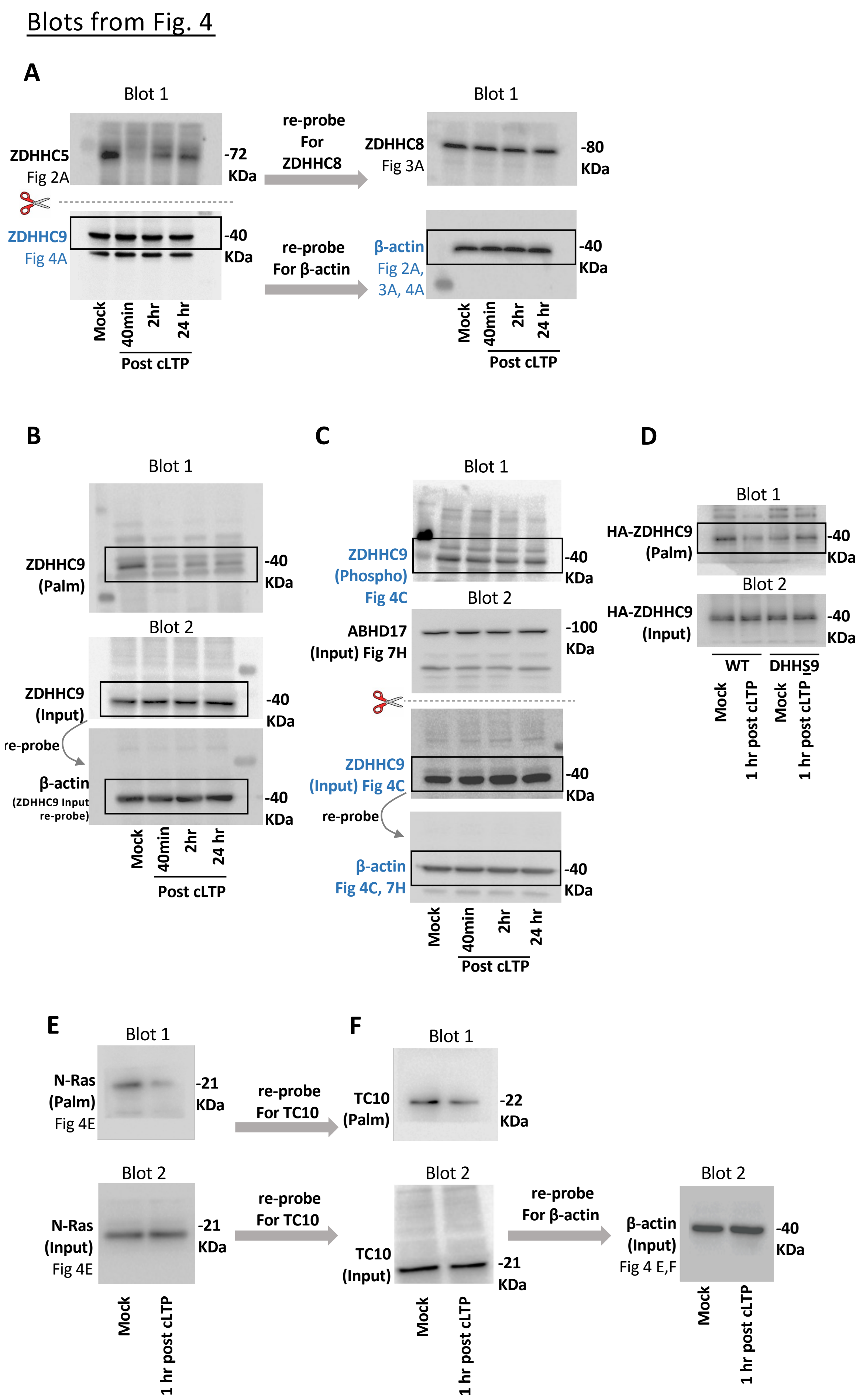

Journal of Cell Science • Supplementary information

Blots from Fig. 5

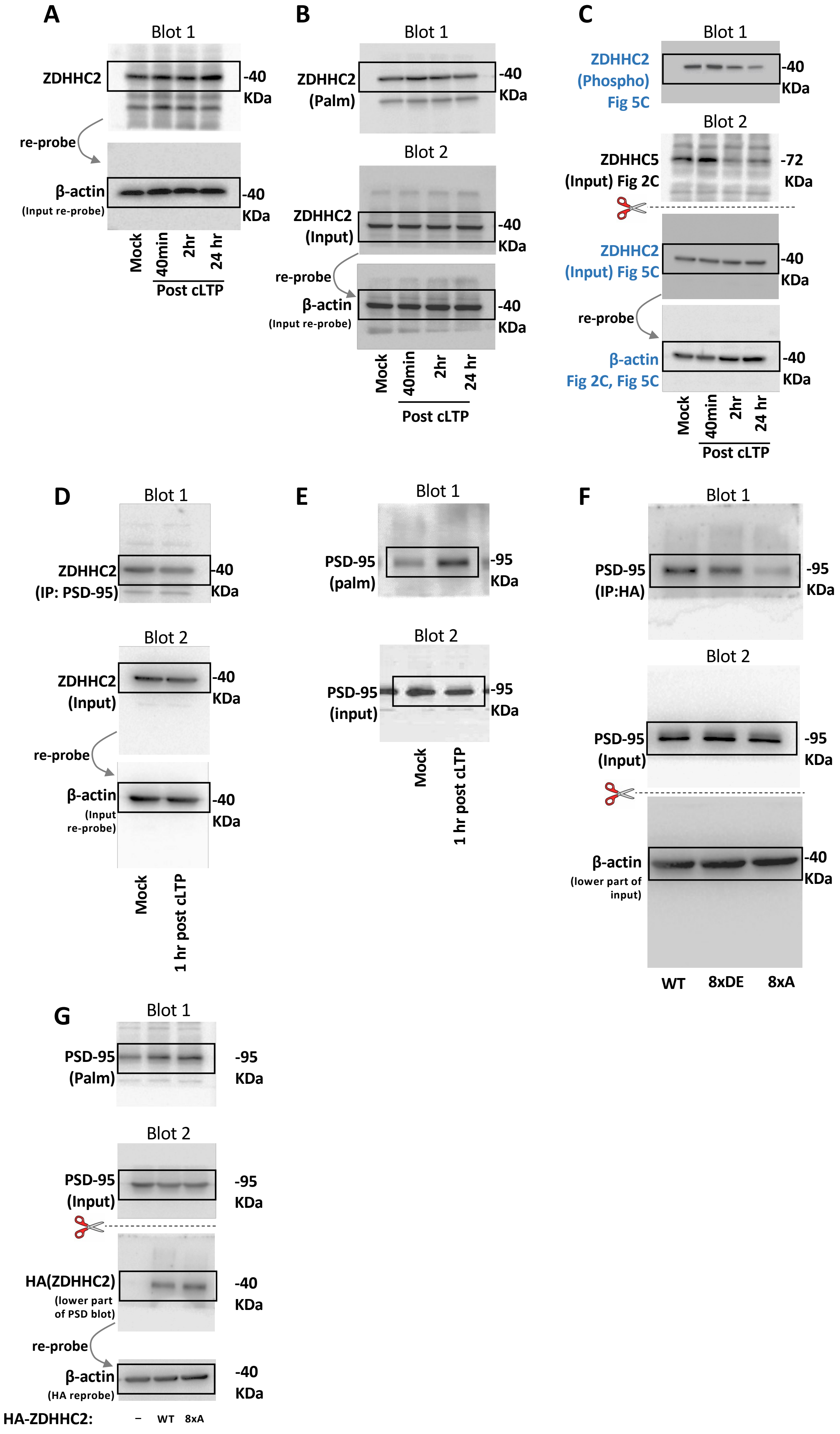

Journal of Cell Science • Supplementary information

Blots from Fig. 6

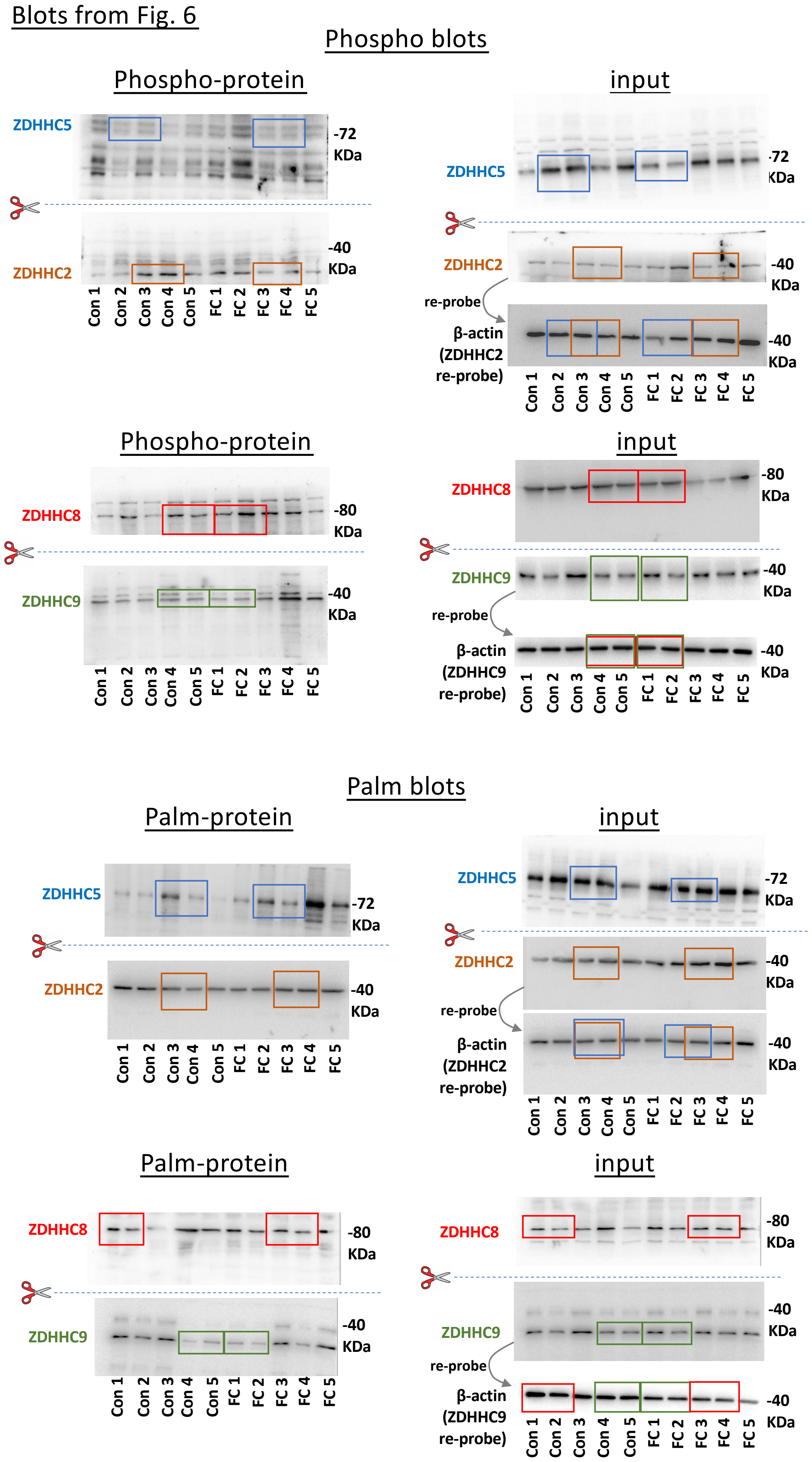

Journal of Cell Science • Supplementary information

Blots from Fig. 7

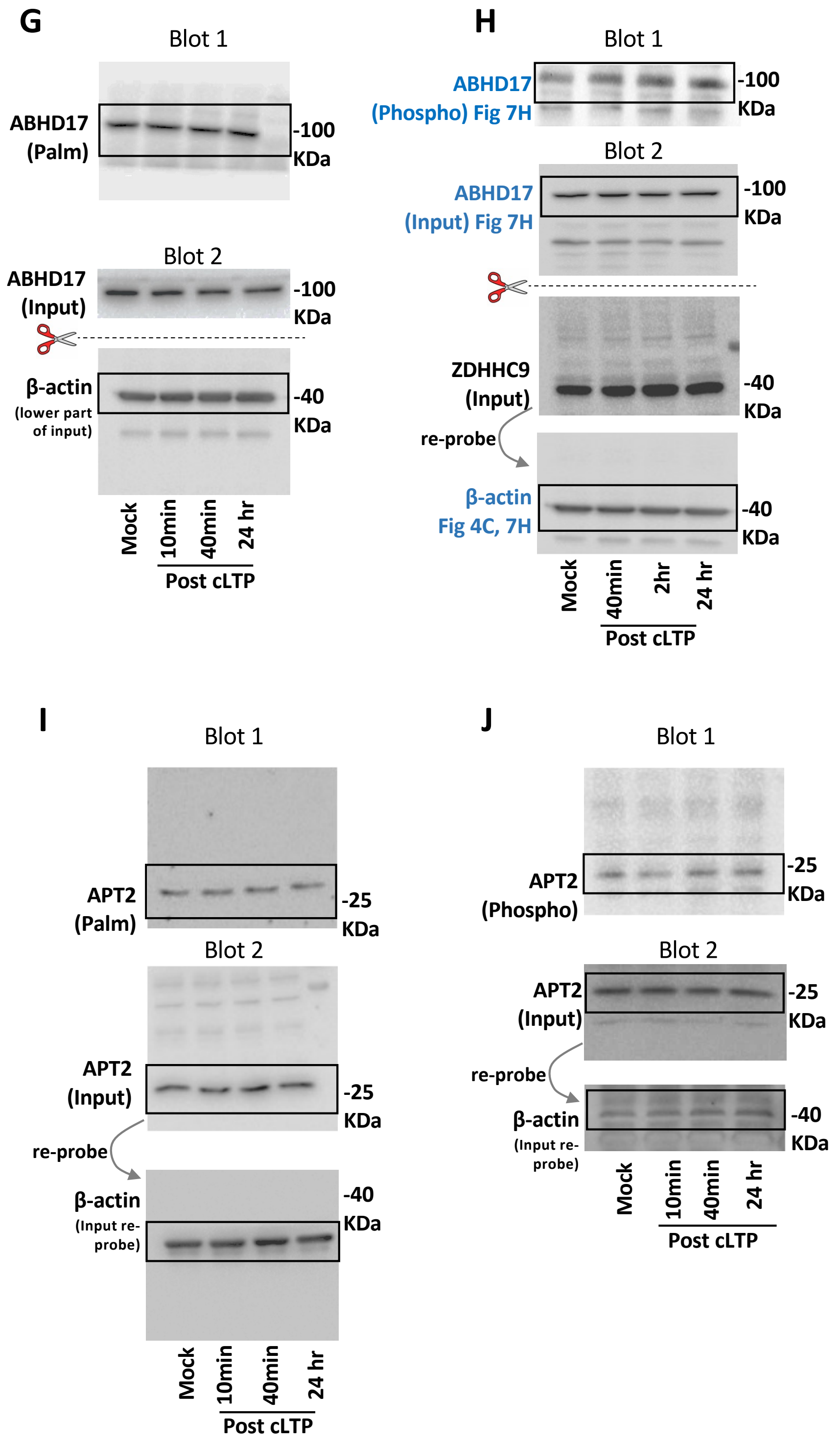

Journal of Cell Science • Supplementary information

Table S1. Primers used for qRT-PCR

Primer for	Forward primer (5' to 3')	Reverse primer (5' to 3')
**ZDHHC1**	GCT CAC CAC CTA CGA ATA CAT TGT	TGT GGG TCT CCT TTG CTT CCT
**ZDHHC2**	GGT GAA CAA TTG TGT TGG ATT TTC	AAA GGC AGT ACA GCA GAG AGT AAG C
**ZDHHC3**	CCA AAG GAA ATG CCA CTA AAG AG	CAC CTG CCC AGG CTT CAG
**ZDHHC4**	CGA ACG TGT TAT TAC TGC TGC AA	GCA CCT CGA GTT CTT TGG AAA
**ZDHHC5**	CCA AAG AAA GAG AAG ACA ATT GTA ATC A	TTA TCT GCC CAT CTG ACA CTT CTG
**ZDHHC6**	ACA CGC GCC GGT AGG A	CCG AAT GCG AAA CCT GAT G
**ZDHHC7**	TTG GTC TTC CTG TGC CTT GAG	CTG GGT GCC GAA CAT GAC T
**ZDHHC8**	GTG TGT GGC TGG CCT TTT CT	ACC ACG TGG AAG CCA GTG A
**ZDHHC9**	TCT GGA AGT CCT CAT TTG CTT CT	CAA GGA AAG TGT GAA ATC CAG TCA
**ZDHHC11**	TGA TGC AGA CCC GAT TCC A	CTT CCG AGG AGG TAG CTC TTT CT
**ZDHHC12**	GCG TCA ATG GGA AGA GCA A	CAG CAC CAG GAG CAG GAA AG
**ZDHHC13**	GCT CGC AGT GCA GGA ATC A	GTG TTG ACC AAA TCC TGG AAC A
**ZDHHC14**	CAG AAC CAA AGA AGT CAT CAT CAA TG	GGA AGA TCT TGC AGG TGA AAC AG
**ZDHHC15**	GTT AAT AAC TGC ATT GGA TTT TCC AA	AGA CTG TTG TAG CAA TGT ACA GGC A
**ZDHHC16**	CAT GTT GCA TGC TGT CCT CAT	TGT TGA TAT GCC TTT CGA TGC T
**ZDHHC17**	TGC AGG CAA CCA TAG ATA CTT TAT G	AAC ACA ACC ATA AAT CAT CCA GCA
**ZDHHC18**	CTG GCC ATC CCC ATC ATC	TCT GCA GGA GAC AGC TCA TGA C
**ZDHHC19**	TCT TTC CCG CGG TCA CA	TGA GCG AGA CGA GAC TGA AGA A
**ZDHHC20**	AAA TCA ACC TTT TCC TAT CAA ACC A	CAC TGA GAT TCA CTG TCC AAC AAA C
**ZDHHC21**	TGG GTT GGC AGG CAG TTT	TCA ACA ACA AAG TGA ATC CGA AGA
**ZDHHC22**	GGC AAG CAC TCC AGC CAT T	GTG CTG TGG GCT TAC GTT CA
**ZDHHC23**	CGC TGA CCT TGA ACA CCA TCT	AGG ACA GTA GAA GAG GGC TGT GA
**ZDHHC24**	CCA CCA CTG TCG CCT ATT GG	CAC AGA AAG GGC CGG TAA TTA T
**ABHD17A**	TCC TGT ATG GCC AGA GCA TTG	CAC TCA TAA CGT GAC GCC AGA T
**ABHD17B**	ACC TCG CTG CTC GGT ATG A	TCC TGA GGT CAA AGG AGA ATG AA
**ABHD17C**	GCA GCC GTC ATC CTC CAT	TCT GGA AAA GCA ACA CGC AAT

Journal of Cell Science • Supplementary information
