# Supplementary material for: Activity-dependent post-translational regulation of palmitoylating and depalmitoylating enzymes in the hippocampus
Source: J Cell Sci. 2023 Apr 11;136(7):jcs260629. doi: 10.1242/jcs.260629 (PMC10113885; doi:10.1242/jcs.260629)
Supplement: Supplementary file 1 [file joces-136-260629_review_history.pdf]

Journal of Cell Science

Activity-dependent post-translational regulation of palmitoylating and depalmitoylating enzymes in the hippocampus

Danya Abazari, Angela R. Wild, Tian Qiu, Bryan C. Dickinson and Shernaz X. Bamji
DOI: 10.1242/jcs.260629

Editor: Giampietro Schiavo

Review timeline
Original submission: 13 September 2022
Editorial decision: 18 October 2022
First revision received: 13 January 2023
Accepted: 20 January 2023

Original submission

First decision letter

MS ID#: JOCES/2022/260629

MS TITLE: Activity-dependent post-translational regulation of palmitoylating and de-palmitoylating enzymes in the hippocampus

AUTHORS: Danya Abazari, Angela R Wild, Tian Qiu, Bryan C Dickinson, and Shernaz X Bamji
ARTICLE TYPE: Research Article

We have now reached a decision on the above manuscript.

To see the reviewers' reports and a copy of this decision letter, please go to: https://submit-jcs.biologists.org and click on the 'Manuscripts with Decisions' queue in the Author Area. (Corresponding author only has access to reviews.)

As you will see, the reviewers gave favourable reports but raised some critical points that will require amendments to your manuscript. I hope that you will be able to carry these out adn Look forward to receive an amended version of your manuscript.

Please ensure that you clearly highlight all changes made in the revised manuscript. Please avoid using 'Tracked changes' in Word files as these are lost in PDF conversion.

I should be grateful if you would also provide a point-by-point response detailing how you have dealt with the points raised by the reviewers in the 'Response to Reviewers' box. Please attend to all of the reviewers' comments. If you do not agree with any of their criticisms or suggestions please explain clearly why this is so.

Reviewer 1

Advance summary and potential significance to field

The manuscript by Abazari et al explores the important question of the mechanism underlying activity-dependent changes in palmitoylation. It is established that many key proteins show activity dependent changes in palmitoylation status and the study sets out to understand the basis for this. It is clearly shown by qPCR analysis that there are not rapid changes in mRNA expression levels of

© 2023. Published by The Company of Biologists under the terms of the Creative Commons Attribution License (https://creativecommons.org/licenses/by/4.0/).

any of the 23 zDHHC palmitoylating enzymes, thus the authors propose a role for post-translational regulation. There are several interesting findings reported in the manuscript including the following responses to cLTP: decreased phosphorylation of zDHHC2 and reduced association with PSD95, decreased palmitoylation and autoacylation (surrogate of activity) of zDHHC9, and increased phosphorylation of a polo-box domain in zDHHC5 associated with enhanced degradation of this enzyme (interestingly this was not seen with the related zDHHC8 enzyme). Some of these changes were also seen in a mouse model of contextual fear conditioning.

Finally no changes were seen looking at ABHD17 and APT2.

The study is well performed. All results are appropriately quantified and analysed. The results are interesting and identify new pathways/mechanisms potentially linked to changes in palmitoylation in response to synaptic activity. There are a couple of areas where the discussion could be extended (see below).

Comments for the author

MINOR SUGGESTIONS

1.The findings that zDHHC8 does not show the same phosphorylation and degradation pattern as zDHHC5 despite also having a polo-box like domain is interesting. Although the difference between zDHHC5 and zDHHC8 is given some attention in the Discussion section, the authors could discuss further if the polo-box domain is actually a substrate for polo kinase- has this been shown? Or have the residues in this domain been shown to be phosphorylated previously?

2.The decrease in active site palmitoylation of zDHHC9 is consistent with a loss of binding to GOLGA7. What is known about phosphorylation of GOLGA7? How does GOLGA7 interact with zDHHC9, is this known? The discussion of these points could be extended a little.

Reviewer 2

Advance summary and potential significance to field

The post-translational modification known as palmitoylation plays a significant role in synaptic activity. This PTM cycle is mediated by palmitoyltransferases that add the modification, and thioesterases that can remove it. It has previously been demonstrated that the palmitoylation of synaptic proteins is dynamically regulated, however the mechanism behind this process is not known.

Following induction of synaptic activity using a LTP, they quantified mRNA transcripts and found no changes in any palmitoyltransferase after 40 or 120 mins. After 24hrs, they found reduced expression of DHHC2, DHHC8 and DHHC22, and an increase in DHHC11. They conclude that regulation of synaptic activity is unlikely due to changes in mRNA levels.

Although at least 23 palmitoyltransferases exist, the authors have focused on a subset, primarily due to available tools to study them. Indeed, the authors screened a number of antibodies to various palmitoyltransferases. Using this information, they followed up on DHHC2, DHHC5, DHHC8 and DHHC9.

In Figure 2, the authors show that DHHC5 expression in reduced following LTP treatment, while the amount of palmitoylation and phosphorylation increases. This increase in phosphorylation results in the degradation of DHHC5. The author also identified the kinases involved in this process.

In Figure 3, the authors found no changes to DHHC8 expression, palmitoylation or phosphorylation after LTP treatment.

In Figure 4, the authors show that the expression and phosphorylation of DHHC9 do not change, but the palmitoylation of the protein was significantly reduced. They further demonstrate that known substrates of DHHC9, NRAS and TC10, had reduced palmitoylation 1 hour post-LTP treatment.

© 2023. Published by The Company of Biologists under the terms of the Creative Commons Attribution License (https://creativecommons.org/licenses/by/4.0/).

In figure 5, the authors found that DHHC2 expression and palmitoylation levels were not changed following LTP treatment, but found a significant reduction if DHHC2 phosphorylation and determined the role of this change in phosphorylation on a substrate of DHHC2, PSD-95. Surprisingly, they found that phosphorylation affected the association between DHHC2 and PSD-95, which lead to an increase in PSD-95 palmitoylation.

The authors have also confirmed their in vitro observations in in vivo.

Finally, they wanted to explore the enzymes that have been shown to remove palmitoylation for target proteins. They found no effect after LTP stimulation, and concluded that regulation of palmitoylation in this case, suggesting that the palmitoyltransferases appear to be the main regulators.

Overall, the authors have presented data demonstrating that external stimuli can regulate the activity of palmitoyltransferases and that this process can be regulated via phosphorylation. Furthermore, their data points to a role of the palmitoyltransferases as a key regulator in this process, with a less significant role of de-palmitoylating enzymes.

Comments for the author

The manuscript contains data on a few palmitoyltrasferases and de-palmitoylating enzymes. Although the data is generally solid and well presented, I find the study in certain aspects a bit premature. For example, in Figure 2, is the fraction of DHHC5 that is palmitoylated also phosphorylated? Does blocking palmitoylation increase, or decrease, the fraction of phosphorylated DHHC5? Is palmitoylated, or non-palmitoylated DHHC5 ubiquitinated? I think further characterization of the interplay between palmitoylation and phosphorylation would add significantly to the manuscript.

Figure 6: In panel A, the conclusion is a decrease in DHHC5 expression, but no change in phosphorylation. I think it would be appropriate to include a loading control in these blots. Based on my observations, the representative blot shows constant levels of phosphorylation, with a decrease in expression. The quantification supports the conclusion. Could the authors supply a more representative blot.

<u>Reviewer 3</u>

Advance summary and potential significance to field

Several studies have shown that many synaptic proteins are dynamically palmitoylated in response to synaptic activity and that palmitoylation of some of these proteins is essential for the recruitment/retention of AMPA receptors at the postsynaptic membrane and synaptic strengthening. Here the authors have sought to identify how synaptic activity controls the enzymes (PATs and depalmitoylation enzymes) that regulate palmitoylation levels to better understand how synaptic activity results in changes in palmitoylation. They have established that synaptic activity does not regulate deplamitoyating enzymes but does regulate the abundance, palmitoylation and phosphorylation of a subset of PATs. They uncovered a novel mechanism that regulates the abundance of DHHC5 involving phosphorylation by Cdk5 and Plk2 leading to DHHC5 degradation. They also find that DHHC2 phosphorylation is associated with reduced substrate interaction but increased substrate palmitoylation. This manuscript represents a significant advance in understanding the mechanism by which synaptic activity leads to changes in synaptic palmitoylation.

Comments for the author

The authors began by assessing whether the transcription of PATs and depalmitoylating enzymes is regulated by synaptic activity and found that, by in large, activity-dependent regulation of these enzymes does not occur at the transcriptional level. They found that cLTP induces a rapid downregulation of DHHC5 protein levels accompanied by an upregulation of DHHC5 palmitoylation and phosphorylation. They identified a Polo box motif on DHHC5 and determined that phosphorylation at this motif was increased by cLTP and regulated DHHC5 degradation as assessed

© 2023. Published by The Company of Biologists under the terms of the Creative Commons Attribution License (https://creativecommons.org/licenses/by/4.0/).

using a phospho-dead version of DHHC5. Overexpression of PLK2 also reduced DHHC5 abundance confirming this further. Overexpression of Cdk5 (and its activator) P35 primed DHHC5 for phosphorylation by PLK2, thereby establishing a mechanism for regulating DHHC5 stability by this signalling cascade.

They also tested DHHC9 and found the cLTP does not affect DHHC9 stability but did reduce its active site palmitoylation and the downstream palmitoylation of one of its substrates. In Figure 4D, the authors show that palmitoylation of the DHHC motif of DHHC9 is reduced upon cLTP. Do the authors think that this is due to a reduction in auto-palmitoylation of DHHC9, or is it mediated by a deplamitoylating enzyme?

A similar analysis of DHHC2 stability, palmitoylation and phosphorylation revealed a decrease in the phosphorylation of DHHC2 in response to cLTP treatment. This was associated with a reduction in DHHC2-PSD-95 binding and a contradictory increase in PSD-95 palmitoylation. Have the authors explored possible compensation by other PATs that could lead to increased palmitoylation of PSD-95 in response to cLTP treatment? Wouldn't this be a more obvious explanation instead of the proposed changes in the kinetics of DHHC2? What kinase(s) phosphorylates DHHC2? A discussion of candidate kinases based on a sequence motif analysis would be useful to include.

The authors sought to validate the changes in PAT abundance, palmitoylation and phosphorylation they observed in vitro with a set of in vivo experiments using fear conditioning. Whilst some of the changes were mirrored in vivo, it is concerning that the decrease in abundance of DHHC5 was not accompanied by an increase in DHHC5 phosphorylation. This calls into question the model proposed in Fig 2K. Given the stimulation paradigms in vitro and in vivo both result in the degradation of DHHC5, the mechanism in Fig 2K must be different or at least more complicated in vivo.

Minor points
In Figure 2C, the levels of DHHC5 in the input do not appear to have been normalised for loading.
The DHHC2 phospho-mimetic/phospho-dead mutants have 8 mutated phosphorylation sites, which could cause significant structural; changes to the protein. The authors should clarify if this part of the protein is required for binding to PSD-95.
The authors propose that the reduction in the interaction between DHHC2 and PSD-95, which is accompanied by an increase in palmitoylation of PSD-95 in response to cLTP, is potentially due to an increase in DHHC2 enzyme kinetics. Is there any precedent for this established for any PAT?

First revision

Author response to reviewers' comments

We have now attached a point-by-point response to reviewers. In general, none of the reviewers expressed any concerns regarding the quality or interpretation of the data, and no additional experiments were specifically requested. Reviewers did pose a number of questions to further flesh out the molecular mechanisms underlying activity-induced post-translational modification of ZDHHC enzymes. However, each of these could be addressed in the Discussion, and we have now highlighted the more fertile avenues for future research as brought up by reviewers. We have also included loading controls in Figure 6 at the reviewer's request.

REVIEWER 1
The study is well performed. All results are appropriately quantified and analysed. The results are interesting and identify new pathways/mechanisms potentially linked to changes in palmitoylation in response to synaptic activity. There are a couple of areas where the discussion could be extended (see below).

We thank the reviewer for their overall positive assessment of our manuscript.

MINOR SUGGESTIONS:
1. The findings that zDHHC8 does not show the same phosphorylation and degradation pattern as zDHHC5 despite also having a polo-box like domain is interesting. Although the difference between

© 2023. Published by The Company of Biologists under the terms of the Creative Commons Attribution License (https://creativecommons.org/licenses/by/4.0/).

zDHHC5 and zDHHC8 is given some attention in the Discussion section, the authors could discuss further if the polo-box domain is actually a substrate for polo kinase- has this been shown? Or have the residues in this domain been shown to be phosphorylated previously?

We could not find any study that demonstrates that ZDHHC8 can be phosphorylated by polo kinase. However, we found that residues S573 and T578 of the ZDHHC8 Polo box were detected to be phosphorylated in a proteomic study (www.phosphosite.org; PMID: 22499769).

We have updated the Discussion and added the italicized portion to the following statement (page 13): "ZDHHC5 contains many regulatory motifs that are also found in ZDHHC8, including a C-terminal PDZ binding domain (EISV; Thomas et al., 2012), tyrosine endocytic motif (YDNL; Brigidi et al., 2015) and a Polo-box like domain *(ZDHHC8 putative polo box sequence: DSGVYDT), which can be phosphorylated at S573 and T578 according to phospho-proteomic database www.phosphosite.org. It is therefore possible that phospho-regulation of the ZDHHC8 polo box could be induced by stimuli other than cLTP."*

2. The decrease in active site palmitoylation of zDHHC9 is consistent with a loss of binding to GOLGA7. What is known about phosphorylation of GOLGA7? How does GOLGA7 interact with zDHHC9, is this known? The discussion of these points could be extended a little.

The reviewer is correct that there are a number of mechanisms by which cLTP could lead to a decrease in ZDHHC9 palmitoylation, including a decrease in GOLGA7 binding. To our knowledge, the binding site for GOLGA7 on ZDHHC9 (or yeast homologs Erf4 on Erf2), has not been precisely mapped, but could potentially lie within the ZDHHC9 C-terminal tail (PMID: 22904317). GOLGA7 is phosphorylated at only one Ser/Thr residue (Thr28) and two Tyr residues (Tyr54 and Tyr76) according to www.phosphosite.org. However, it is unclear if these phospho-sites are regulated by activity, or alter interactions between ZDHHC9 and GOLGA7.

Previous studies have shown that disrupting the interaction between yeast homologs of ZDHHC9 (Erf2) and GOLGA7 (Erf4) reduces the stability of Erf2, however we did not observe a decrease in ZDHHC9 total protein following cLTP, suggesting that alternative mechanisms might alter ZDHHC9 active site palmitoylation other than a decreased interaction between GOLGA7 and ZDHHC9. We have edited the Discussion section on ZDHHC9 to include these ideas.

We now state in the Discussion *(page 14)*: *"It is unclear how neuronal activity may lead to decreased ZDHHC9 palmitoylation. It is possible that cLTP could alter ZDHHC9 accessibility to palmitoylating/depalmitoylating enzymes. As the decreased palmitoylation of ZDHHC9 appears to occur within the catalytic domain of the protein, it is possible that cLTP could alter the interaction of ZDHHC9 with its cofactor, GOLGA7, leading to a decrease in ZDHHC9 autopalmitoylation. However, we did not observe a concurrent decrease in ZDHHC9 total protein that might be expected if the interaction was disrupted (Swarthout et al., 2005), indicating that a mechanism other than a decrease in GOLGA7 binding may be responsible for decreased ZDHHC9 palmitoylation. Other mechanisms that alter ZDHHC9 autopalmitoylation within the active site may also be responsible."*

REVIEWER 2

Figure 2, is the fraction of DHHC5 that is palmitoylated also phosphorylated? Does blocking palmitoylation increase, or decrease, the fraction of phosphorylated DHHC5? Is palmitoylated, or non- palmitoylated DHHC5 ubiquitinated? I think further characterization of the interplay between palmitoylation and phosphorylation would add significantly to the manuscript.

The cross-talk between protein palmitoylation and phosphorylation is a very interesting question and one that certainly warrants further exploration. However, we submit that this is the subject of an entirely separate study and does not further support the main conclusion of this study. Indeed, a recent publication has entirely been dedicated to

© 2023. Published by The Company of Biologists under the terms of the Creative Commons Attribution License (https://creativecommons.org/licenses/by/4.0/).

studying the crosstalk of palmitoylation and phosphorylation on synapsin1 (PMID: 36097267). To further stress the complexity of what is being requested, in addition to activity-induced changes in the phosphorylation of ZDHHC5 Polo-box domain and tyrosine endocytic motif (PMID: 26334723), ZDHHC5 has been reported to have three additional phosphorylated sites (Ser380, Ser432 and Ser 621) that can be modified in response to activity (PMID: 34433048). Therefore, a comprehensive study of the interplay between activity-dependent palmitoylation, phosphorylation of the various serines/threonines and tyrosines on ZDHHC5, and ubiquitination would require a considerable amount of work.

Figure 6: In panel A, the conclusion is a decrease in DHHC5 expression, but no change in phosphorylation. I think it would be appropriate to include a loading control in these blots. Based on my observations, the representative blot shows constant levels of phosphorylation, with a decrease in expression. The quantification supports the conclusion. Could the authors supply a more representative blot.

The reviewer is correct that the blots in Fig 6A show a decrease in total ZDHHC5 protein, and no change in phosphorylation. However, because we normalized the phospho-ZDHHC5 to ZDHHC5 input, this resulted in a trend towards an increase in the phosphorylation of the remaining ZDHHC5, which was not significant. We therefore think that the blots included in the figure are appropriate.

We have now included the beta-actin reprobes for all blots in Figure 6. We agree with the reviewer this is a necessary addition to the figure. Please note that β-actin loading controls are duplicated in some panels as individual blots were cut in half and probed for 2 ZDHHCs per blot. Cut blots were probed for either ZDHHC2 (bottom half)/ZDHHC5 (top half) or ZDHHC9 (bottom half)/ZDHHC8 (top half). These details have been included in the figure legend for clarification.

REVIEWER 3
They also tested DHHC9 and found the cLTP does not affect DHHC9 stability but did reduce its active site palmitoylation and the downstream palmitoylation of one of its substrates. In Figure 4D, the authors show that palmitoylation of the DHHC motif of DHHC9 is reduced upon cLTP. Do the authors think that this is due to a reduction in auto-palmitoylation of DHHC9, or is it mediated by a depalmitoylating enzyme?

The reviewer raises an interesting question. Given that we don't know what enzymes depalmitoylate ZDHHC9, it would be very difficult to resolve these two possibilities and any additional commentary would just be guesswork. However, we have expanded the discussion to highlight these two possibilities and also expand the discussion around GOLGA7. In the discussion we state:

(page 14): *"It is unclear how neuronal activity may lead to decreased ZDHHC9 palmitoylation. It is possible that cLTP could alter ZDHHC9 accessibility to palmitoylating/depalmitoylating enzymes. As the decreased palmitoylation of ZDHHC9 appears to occur within the catalytic domain of the protein, it is possible that cLTP could alter the interaction of ZDHHC9 with its cofactor, GOLGA7, leading to a decrease in ZDHHC9 autopalmitoylation. However, we did not observe a concurrent decrease in ZDHHC9 total protein that might be expected if the interaction was disrupted* (Swarthout et al., 2005)*, indicating that a mechanism other than a decrease in GOLGA7 binding may be responsible for decreased ZDHHC9 palmitoylation"*

A similar analysis of DHHC2 stability, palmitoylation and phosphorylation revealed a decrease in the phosphorylation of DHHC2 in response to cLTP treatment. This was associated with a reduction in DHHC2-PSD-95 binding and a contradictory increase in PSD-95 palmitoylation. Have the authors explored possible compensation by other PATs that could lead to increased palmitoylation of PSD-95 in response to cLTP treatment? Wouldn't this be a more obvious explanation instead of the proposed changes in the kinetics of DHHC2?

We agree with the reviewer that further discussion is required to explain why we suggest that activity-dependent dephosphorylation of ZDHHC2 may impact ZDHHC2 kinetics. While

© 2023. Published by The Company of Biologists under the terms of the Creative Commons Attribution License (https://creativecommons.org/licenses/by/4.0/).

possible compensation by other PATs may explain our result, the Fukata lab has done an extensive study showing that the ZDHHC2/15 subfamily plays a major role in activity-sensitive PSD-95 palmitoylation (PMID: 19596852). However, given that the expression of ZDHHC15 is much lower in the hippocampus (PMID: 19596852; 35819139), ZDHHC2 is thought to be the primary activity-induced PAT for PSD-95. We therefore think it most likely that changes in ZDHHC2 phosphorylation are coupled to changes in PSD-95 palmitoylation. While we speculate that this involves changes in ZDHHC2 kinetics, further work is needed to fully delineate the mechanism.

We have added the following to the manuscript (page 15): "C*ompensation by other ZDHHC enzymes could explain our result; however previous work has found PSD-95 to be a substrate for a limited number of ZDHHC enzymes including ZDHHC2, ZDHHC3, ZDHHC7 and ZDHHC15, with only ZDHHC2/ZDHHC15 participating in activity-dependent PSD-95 palmitoylation (Noritake et al., 2009). However, given that the expression of ZDHHC15 is very low in the hippocampus, ZDHHC2 is thought to be the primary palmitoylating enzyme for PSD-95 (Wild et al., 2022; Noritake et al., 2009). While further work is required to fully explain the mechanism, it is likely that ZDHHC2 dephosphorylation is mediating the increase in PSD-95 palmitoylation.*"

What kinase(s) phosphorylates DHHC2? A discussion of candidate kinases based on a sequence motif analysis would be useful to include.

We have added the following to the discussion in line with the reviewer's request (page 15):

"It will be necessary in future to identify the kinases and phosphatases that modify ZDHHC2. Using prediction software KinasePhos3 (Ma et al., 2022) we found that the ZDHHC2 C-terminal has a very strong probability of being a substrate for a number of kinases, including PKD1, AMPK1, CAMK2A and PKA. Notably, both CAMK2A and PKA are known to be highly responsive to LTP stimuli indicating that these enzymes may play a role in dynamic phosphorylation of ZDHHC2 (Woolfrey and Dell'Acqua, 2015)."

The authors sought to validate the changes in PAT abundance, palmitoylation and phosphorylation they observed in vitro with a set of in vivo experiments using fear conditioning. Whilst some of the changes were mirrored in vivo, it is concerning that the decrease in abundance of DHHC5 was not accompanied by an increase in DHHC5 phosphorylation. This calls into question the model proposed in Fig 2K. Given the stimulation paradigms in vitro and in vivo both result in the degradation of DHHC5, the mechanism in Fig 2K must be different or at least more complicated in vivo.

We submit that the differences in posttranslational modifications of ZDHHC enzymes observed following cLTP treatment of hippocampal cultures and fear conditioning *in vivo*, is most readily explained by differences in the two preparations and the ability of the assay to detect differences in phosphorylation. While we do see a significant decrease in ZDHHC5 levels and only a trending increase in ZDHHC5 phosphorylation *in vivo*, we do not think it negates the model proposed in Fig 2K. We submit that because whole hippocampal lysates are composed of multiple cell types and not just neurons, there may be an issue of sensitivity in observing changes in ZDHHC5 phosphorylation (i.e. ZDHHC5 in non-neuronal cells and different neuron subtypes may occlude our ability to detect significant increases in ZDHHC5 phosphorylation).

Minor points

In Figure 2C, the levels of DHHC5 in the input do not appear to have been normalised for loading.

We apologize for the confusion. In the Methods we had written (page 20-21): *"For western blot analysis, the protein of interest input was first normalized to β-actin as a loading control. For Acyl-Rac palmitoylation and phospho-protein assays, the amount of palmitoylated or phospho- protein was then normalized to the β-actin normalized input"*. This is how we did the analysis. In our original submission figure legends, we had

© 2023. Published by The Company of Biologists under the terms of the Creative Commons Attribution License (https://creativecommons.org/licenses/by/4.0/).

erroneously written: “*Phosphorylated ZDHHC5 values in graph derived from ZDHHC5 ‘phospho’ normalized to ZDHHC5 ‘input’*”.

We have now corrected this to “*Phosphorylated ZDHHC5 values in graph derived from ZDHHC5 ‘phospho’ normalized to ZDHHC5 ‘input’ and β-actin loading control*”, and have edited all other instances in the figure legends that describe data normalization accordingly.

The DHHC2 phospho-mimetic/phospho-dead mutants have 8 mutated phosphorylation sites, which could cause significant structural; changes to the protein. The authors should clarify if this part of the protein is required for binding to PSD-95.

While we could not find any study that precisely mapped the binding site of PSD-95 on ZDHHC2, the mutated Ser and Thr residues in the HA-ZDHHC2-8xDE phosphomimetic and HA-ZDHHC2- 8xA phosphodead mutants are all located within the distal C-terminal (330-366 aa), which is predicted to be predominantly unstructured (https://iupred3.elte.hu/).

We have added the following to the Discussion (*page 15)*:
“*Although the binding site of PSD-95 on ZDHHC2 has not been precisely mapped, the mutated Ser and Thr residues in the HA-ZDHHC2-8xA phospho-dead mutant are unlikely to cause structural changes in the protein that might disrupt PSD-95 binding, as these residues are located within the distal C-terminal (330-366 aa), which is predicted to be predominantly unstructured (https://iupred3.elte.hu/)*”.

The authors propose that the reduction in the interaction between DHHC2 and PSD-95, which is accompanied by an increase in palmitoylation of PSD-95 in response to cLTP, is potentially due to an increase in DHHC2 enzyme kinetics. Is there any precedent for this established for any PAT?

We do not know of a precedent for this type of regulation of any PAT, and as such this is offered as a speculative interpretation of our results. We have greatly expanded the discussion section on ZDHHC2 in response to a number of reviewer comments and believe that this section is now strengthened with the addition of a number of alternative potential regulatory mechanisms.

We write (page 15):
“*Compensation by other ZDHHC enzymes could also explain our result, however previous work has found PSD-95 to be a substrate for a limited number of ZDHHC enzymes including ZDHHC2, ZDHHC3, ZDHHC7 and ZDHHC15, with only ZDHHC2/ZDHHC15 participating in activity-dependent PSD-95 palmitoylation (Noritake et al., 2009). However, given that the expression of ZDHHC15 is very low in the hippocampus, ZDHHC2 is thought to be the primary palmitoylating enzyme for PSD- 95 (Wild et al., 2022; Noritake et al., 2009). Although further work is required to fully explain the mechanism, it is likely that ZDHHC2 dephosphorylation is mediating the increase in PSD-95 palmitoylation.*

The binding site of PSD-95 on ZDHHC2 has not been precisely mapped, however the mutated Ser and Thr residues in the HA-ZDHHC2-8xA phosphodead mutant are unlikely to cause structural changes in the protein that might disrupt PSD-95 binding, as these residues are located within the distal C-terminal (330-366 aa), which is predicted to be predominantly unstructured (https://iupred3.elte.hu/).

It will be necessary in future to identify the kinases and phosphatases that modify ZDHHC2. Using prediction software KinasePhos3 (Ma et al., 2022) we found that the ZDHHC2 C-terminal has a very strong probability of being a substrate for a number of kinases, including PKD1, AMPK1, CAMK2A and PKA. Notably, both CAMK2A and PKA are known to be highly responsive to LTP stimuli indicating that these enzymes may play a role in dynamic phosphorylation of ZDHHC2 (Woolfrey and Dell’Acqua, 2015).”

© 2023. Published by The Company of Biologists under the terms of the Creative Commons Attribution License (https://creativecommons.org/licenses/by/4.0/).

Second decision letter

MS ID#: JOCES/2022/260629

MS TITLE: Activity-dependent post-translational regulation of palmitoylating and de-palmitoylating enzymes in the hippocampus

AUTHORS: Danya Abazari, Angela R Wild, Tian Qiu, Bryan C Dickinson, and Shernaz X Bamji
ARTICLE TYPE: Research Article

I am happy to tell you that your manuscript has been accepted for publication in Journal of Cell Science, pending standard ethics checks.

Reviewer 1

Advance summary and potential significance to field

As per previous version

Comments for the author

Thank you for additions made in response to my suggestions/queries

Reviewer 2

Advance summary and potential significance to field

This was mentioned in my first review of the manuscript

Comments for the author

This is a revised manuscript from a previous review.
The authors have addressed my concerns, and I support publication of this manuscript.

Reviewer 3

Advance summary and potential significance to field

Several studies have shown that many synaptic proteins are dynamically palmitoylated in response to synaptic activity and that palmitoylation of some of these proteins is essential for the recruitment/retention of AMPA receptors at the postsynaptic membrane and synaptic strengthening. Here the authors have sought to identify how synaptic activity controls the enzymes (PATs and depalmitoylation enzymes) that regulate palmitoylation levels to better understand how synaptic activity results in changes in palmitoylation. They have established that synaptic activity does not regulate deplamitoyating enzymes but does regulate the abundance, palmitoylation and phosphorylation of a subset of PATs. They uncovered a novel mechanism that regulates the abundance of DHHC5 involving phosphorylation by Cdk5 and Plk2 leading to DHHC5 degradation. They also find that DHHC2 phosphorylation is associated with reduced substrate interaction but increased substrate palmitoylation. This manuscript represents a significant advance in understanding the mechanism by which synaptic activity leads to changes in synaptic palmitoylation.

Comments for the author

I am satisfied by the responses to my comments and recommend that this manuscript is accepted for publication.

© 2023. Published by The Company of Biologists under the terms of the Creative Commons Attribution License (https://creativecommons.org/licenses/by/4.0/).
